# Prognosis Parameters of Oral Carcinomas Developed in Proliferative Verrucous Leukoplakia: A Systematic Review and Meta-Analysis

**DOI:** 10.3390/cancers13194843

**Published:** 2021-09-28

**Authors:** Miguel Ángel González-Moles, Saman Warnakulasuriya, Pablo Ramos-García

**Affiliations:** 1 Department of Stomatology, School of Dentistry, University of Granada, 18010 Granada, Spain; pramos@correo.ugr.es; 2 Instituto de Investigación Biosanitaria ibs.GRANADA, 18012 Granada, Spain; 3 Faculty of Dentistry, Oral and Craniofacial Sciences, King’s College, London SE1 9RT, UK; saman.warne@kcl.ac.uk

**Keywords:** malignant transformation, meta-analysis, oral cancer, prognosis, proliferative verrucous leukoplakia, systematic review

## Abstract

**Simple Summary:**

Proliferative verrucous leukoplakia is considered by the WHO as an oral potentially malignant disorder that presents the highest tendency to recurrence and malignant transformation rate. However, to date limited evidence-based prognostic data for oral carcinomas developed in patients with proliferative verrucous leukoplakia (PVL-OC) have been published, and these carcinomas probably perform better than conventional oral carcinomas. In this study we present a systematic review and meta-analysis to evaluate the current evidence in relation to the prognosis of oral carcinomas developed in patients PVL-OC.

**Abstract:**

Proliferative verrucous leukoplakia (PVL) is contemplated by the World Health Organization (WHO) as an oral potentially malignant disorder (OPMD) with a high the highest malignant transformation ratio among all OPMD (approximately 50%). Our aim was to evaluate the current evidence in relation to the prognosis of oral carcinoma developed in patients with proliferative verrucous leukoplakia (PVL-OC). We searched PubMed, Embase, Web of Science and Scopus for published studies (upper date limit = June 2021). We evaluated the quality of studies (QUIPS tool). We carried out meta-analyses, examined inter-study heterogeneity through subgroup and meta-regression analyses, and performed sensitivity and small-study effects analyses to test the stability and reliability of results. 23 studies met inclusion criteria (505 patients with PVL, of which 288 developed a total of 504 carcinomas). The meta-analyzed overall mortality rate was 21.29% (pooled proportions [PP] = 95% confidence intervals [CI] = 8.77–36.36) for PVL-OC, clearly lower than the 34.7–50% mortality rate for conventional oral cancer reported in previous studies. In comparison with a single study reporting on conventional oral cancers, mortality was significantly lower for PVL-OC (hazard ratio = 0.29 [95%CI = 0.10–0.89], *p* = 0.03). Univariable meta-regression verified that case series that presented higher proportions of verrucous carcinomas showed a better survival of PVL-OC (*p* = 0.05), but not with higher proportion of oral squamous cell carcinomas (*p* = 0.74). Significant differences were not found for other relevant variables such as follow up period (*p* = 0.44) or multiple tumor development (*p* = 0.74). In conclusion, PVL-OC show favorable prognostic parameters, especially with regard to the mortality rate.

## 1. Introduction

An international seminar convened by the World Health Organization (WHO) Collaborating Center for Oral Cancer in Glasgow (Scotland) in 2020 has reported an update on nomenclature and classification of oral potentially malignant disorders (OPMD) [1]. Proliferative verrucous leukoplakia (PVL) has been defined by this expert group as a distinct form of multifocal oral leukoplakia characterized by having a progressive clinical course, changing clinical, and histopathological features, associated with the highest malignant transformation rate in comparison with other OPMDs. Some members of our research group who participated as experts in the aforementioned consensus meeting, were commissioned to update the information related to the malignant transformation of PVL, which they performed through a systematic review and meta-analysis that reported a rate of malignancy of 43.87% (95%CI = 31.93–56.13) [2], slightly lower than previously published by Iocca et al. (49.5% (CI 26.7–72.4%) [3]. A pertinent fact of our study refers to the wide variability in the proportion of PVL with oral cavity cancer development across the studies assessed in our meta-analysis, ranging between 0 and 100% [4,5] and between 14.29 and 75% [6,7] after the omission of extreme values.

One of the groups that has studied this aspect in greater depth has reported that in its series of 55 patients with PVL, 27 patients developed oral cancer of which 11 patients developed multiple tumors (40.74%) [8]. One might think that PVL, because it is an OPMD with a high rate of malignancy, due to it has a notable tendency to multiple tumor development, in most cases, resistant to all forms of treatment, would lead to the development of carcinomas of poor prognostic evolution. However, to date limited evidence-based prognostic data for oral carcinomas developed in patients with proliferative verrucous leukoplakia (PVL-OC) have been published and one series that analyze prognostic parameters indicate that these carcinomas probably perform better than conventional oral carcinomas [9], with a 5-year mortality rate ranging from 34.70% to 50% [10,11]).

Based on this background, it seems pertinent to evaluate current evidence in relation to the prognosis of PVL-OC, to determine the mortality rate of PVL-OC and to explore the impact of potential covariables on survival (i.e., follow up periods, multiple tumor development, and histology of carcinomas).

## 2. Materials and Methods

Our study was designed complying with high standards criteria developed by Cochrane Handbook for Systematic Reviews of Interventions [12], Cochrane Prognosis Methods Group [13] and Centre for Reviews and Dissemination (CRD)’s guidance for undertaking reviews in health care [14]. MOOSE and PRISMA reporting guidelines were closely followed [15,16].

### 2.1. Protocol

A protocol was a priori designed describing the planned methods of the study, in order to improve the precision and transparency, and to minimize the risk of bias. This study protocol was registered in PROSPERO international prospective register of systematic reviews (www.crd.york.ac.uk/PROSPERO; CRD42021267896 code was assigned) [17]. The protocol also followed PRISMA-P statement [18].

### 2.2. Search Strategy

We searched MEDLINE (accessed through PubMed), Embase, Web of Science and Scopus databases for studies published before June-2021, without lower date limits restriction. Search strategy was designed to maximize sensitivity and performed by combining the following keywords: “proliferative” AND “verrucous” AND “leukoplakia”. Due to the lack of specific thesaurus terms for proliferative verrucous leukoplakia, Emtree and/or MeSH terms were not applied. Reference lists of retrieved studies were also manually searched for additional relevant studies. All references were handled and duplicates eliminated using the software Mendeley v.1.19.8 (Elsevier, Amsterdam, The Netherlands).

### 2.3. Eligibility Criteria

Inclusion criteria: (1) Original primary-level studies published in any language or publication date; (2) Report of mortality and/or clinicopathological parameters of the patients with PVL-OC; (3) For results derived from the same study population, we included the most informative studies; if overlapping populations were suspected, the name of authors, affiliations, treatment centers, and recruitment periods were checked and compared.

Exclusion criteria: (1) Retractions, case reports, reviews, meta-analyses, letters, editorials, personal opinions, comments, meeting abstracts, and book chapters; (2) Preclinical research (animal experimentation and/or in vitro studies); (3) Lack of clinicopathological parameters or survival data; (4) Insufficient data for statistical analysis; (5) Carcinomas developed in anatomical sites other than the oral cavity; (6) overlapping populations (see inclusion criterion No. 3).

### 2.4. Study Selection Process

The eligibility criteria were applied independently by two authors (M.Á.G.-M. and P.R.-G.) in two phases: First, titles and abstracts were screened searching for articles apparently meeting our inclusion criteria; Second, papers were full-text read and excluding the articles not meeting our eligibility criteria. Discrepancies between authors were resolved by consensus. An authors’ agreement score was calculated using Cohen’s kappa (κ) statistic [19], obtaining an almost perfect score (99.20% of agreement, κ = 0.93).

### 2.5. Data Extraction

Two authors (M.Á.G.-M. and P.R.-G.) independently extracted data—filling out a standardized data collection form in Word and Excel (v.16/2018, Microsoft. Redmond, WA)—from the selected articles. Data expressed as order statistics (i.e., medians, interquartile, and/or minimum-maximum ranges) were computed and transformed into means ± standard deviations (SD) using the methods proposed by Luo et al. (2018) and Wan et al. (2014) [20,21]. Means ± SDs from two or more different subgroups were combined into a single dataset if necessary, applying the Cochrane Handbook’s formula [12]. Data was collected on the first author and publication year, country and continent, follow up and recruitment periods, design of study, sample size of PVL-OC, presence of tumor multiple development and number of tumors, tumor subsites, age and sex of patients, tobacco consumption, mortality data of patients, and clinicopathological parameters (histology of carcinomas, differentiation grade of squamous cell carcinomas, clinical stage, T status and/or N status). In one study [22], the prognostic value of the PVL-OC was compared with that of the conventional OC. From these selected studies we attempted to extract data on the outcome overall survival (defined as the time elapsed from date of diagnosis/surgery to date of death by any cause). Disease-free survival or other recurrence parameters were not reported.

### 2.6. Evaluation of Quality and Risk of Bias

Risk of bias (RoB) was assessed in primary-level studies using the Quality in Prognosis Studies-QUIPS tool, supported by *Cochrane Prognosis Methods Group* for prognosis studies [23]. QUIPS considers the following domains: (1) Study participation, (2) Study attrition, (3) Prognostic Factor Measurement, (4) Outcome Measurement, (5) Study confounding, and (6) Statistical Analysis and Reporting [24]. RoB was qualified as low, moderate, or high for each domain. RoB was assessed by two authors (M.A.G.M. and P.R.G.). Discrepancies between authors were resolved by consensus.

### 2.7. Statistical Analysis

Proportions from primary-level studies—expressed as percentages—were calculated by extracting raw numerators (number of patients with PVL-OC and positive parameters, i.e., number of deaths, patients with verrucous or squamous cell carcinomas, well-differentiated squamous cell carcinomas, T1/2, N+, M+ and advanced stage) and denominators (total number of patients with PVL-OC). Their corresponding 95% confidence intervals (CIs) were estimated for primary-studies according to the score-test statistic [25]. The influence of studies with extreme proportions values (i.e., 100%, 0% or close) was minimized by using Freeman–Tukey double-arcsine transformation to stabilize the variance of proportions [26]. The mortality rate and clinicopathological parameters of patients with PVL-OC were meta-analyzed by combining proportions (pooled proportions, PP) and 95% CIs using the inverse-variance method under a random-effects model (based on the Der Simonian and Laird method). Forest plots were constructed for all meta-analyses.

Heterogeneity between studies was checked using the χ^2^ based Cochran’s Q test. Given the low statistical power of this test, *p* < 0.10 was considered significant. We also used Higgins I^2^ statistic to quantify the percentage heterogeneity (considering values of 50–75% as moderate-to-high degree of inconsistency across the studies), which estimates what proportion of the variance in observed effects reflects variation in true effects, rather than sampling error [27,28]. Preplanned stratified meta-analyses (by geographical area) were performed to identify potential sources of heterogeneity for survival parameters. Furthermore, additional univariable meta-regression analyses, using the restricted maximum likelihood (REML) method, were conducted to explore the potential effect of study covariates (follow up period, tumor multiple development, verrucous and oral squamous cell carcinomas) on the mortality of patients with PVL-OC [29]. Taking into consideration the low number of observations for meta-regressions, the *p*-values were recalculated using a permutation test based on Monte Carlo simulations [30] (10,000 permutations series were run to obtain enough precision [31]). Weighted bubble plots were also constructed to graphically represent the fitted meta-regression lines.

Furthermore, the prognostic value of PVL-OC (compared to conventional OC) was analyzed. Due to only one study [22] reporting data for this analysis, meta-analysis could not be performed for this parameter. Hazard ratio (HR) with 95% CI was used to estimate the potential impact of PVL-OC on overall survival. As authors did not explicitly report HR with 95% CIs, we made an estimation using the methods described in Parmar et al. [32] and Tierney et al. [33].

Furthermore, secondary analyses were carried out to test the stability and reliability of our meta-analytical results. Therefore, sensitivity analysis series were carried out to investigate the influence of each primary-level study on the pooled estimates [34], repeating sequentially the meta-analyses, omitting one study at a time (“leave-one-out” method). Finally, small-study effects and potential biases, such as publication bias, were evaluated constructing funnel plots and using the Egger [35] regression test (performing a linear regression of the effect estimates on their standard errors, weighting by 1/[variance of the effect estimate], considering a *p*_Egger_-value < 0.10 as significant). As the absence of small-study effects was confirmed, as advised, additional analyses (e.g., non-parametric Trim & Fill method) were not used to avoid type-I errors (i.e., false-positives missing studies) and subsequent misleading funnel plot asymmetry corrections [36]. Stata software was used for statistical analysis (v.16.1, Stata Corp, College Station, TX, USA).

## 3. Results

### 3.1. Results of the Literature Search

**Figure 1:**
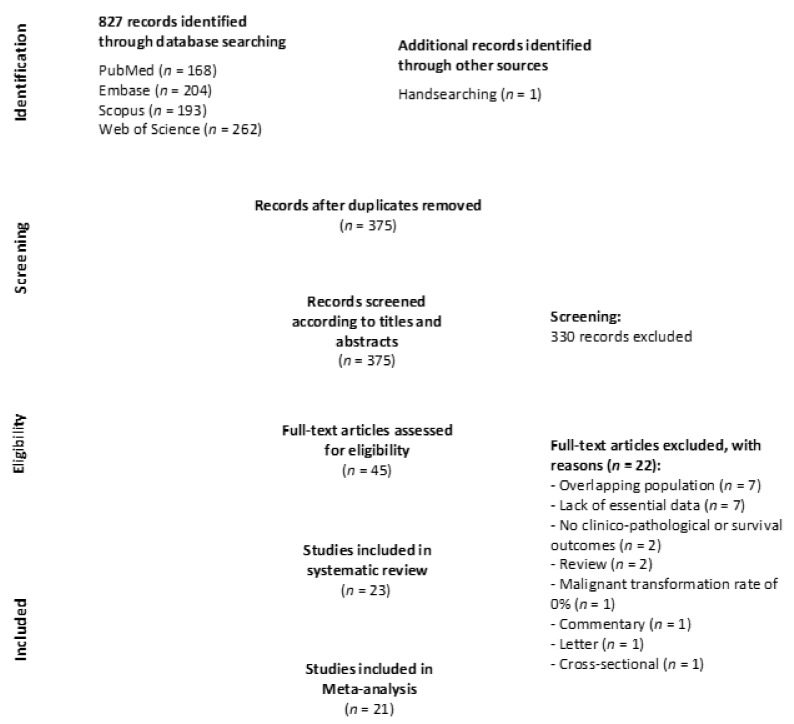
The flow diagram depicts identification and selection process of studies addressing the behavior PVL-OC.

A total of 827 records were identified during the identification and selection process (Figure 1 Flow diagram): 262 from the Web of Science, 204 from Embase, 193 from Scopus, 169 from PubMed, and one after handsearching the reference lists of retrieved studies. After eliminating duplicates, 375 studies were considered potentially eligible. Then, their titles and abstracts were screened and 45 papers selected for full-text reading (22 of them did not meet our eligibility criteria and were excluded; their references and exclusion reasons are listed in the Supplementary Materials, pp. 32–34). Finally, 23 studies were included in the systematic review’s final sample—23 for qualitative evaluation and 21 for quantitative meta-analysis- [5,6,7,22,37,38,39,40,41,42,43,44,45,46,47,48,49,50,51,52,53,54,55].

### 3.2. Study Characteristics

Table 1 summarizes the main characteristics of the selected studies, and Table S1 summarizes in more detail the characteristics of each primary-level study (Supplementary Materials, pp. 3–5). Twenty-three studies published between 1985 and 2021 recruited 543 patients with PVL, of which 288 patients developed a total of 504 oral carcinomas. Nine studies were conducted in Europe (3 in Italy, 3 in Spain, 2 in UK and 1 in France), 9 in North America (all in USA), 3 in Asia (1 each in India, Israel and Malaysia), 1 in South America (Brazil), and 1 was a multicontinent multicentric study (Brazil, USA).

Table 2 exhibits the results of the conducted meta-analyses in the present study. It was feasible to perform meta-analysis for the mortality rate of patients with PVL-OC (183 patients with PVL that developed cancer, enrolled in 14 studies), the proportion of patients with verrucous carcinomas and oral squamous cell carcinomas (255 patients/20 studies, respectively), with well-differentiated oral squamous cell carcinomas (49 patients/8 studies), with a tumor size of T1/2 (13 patients/2 studies), with N+ status (19 patients/3 studies), with M+ status (13 patients; 2 studies), and I/II-clinical stage (46 patients; 3 studies). In addition, the prognosis of these patients (with PVL-OC) was estimated for the overall survival parameter, in comparison to a control group of patients who developed conventional oral carcinomas (without prior history of proliferative verrucous leukoplakia) (60 patients; one study [22]). Finally, several meta-regression analyses were carried out to estimate the potential impact of different variables (i.e., follow up period, tumor multiple development, verrucous and oral squamous cell carcinomas) on the mortality of patients with PVL-OC.

### 3.3. Qualitative Evaluation

**Figure 2:**
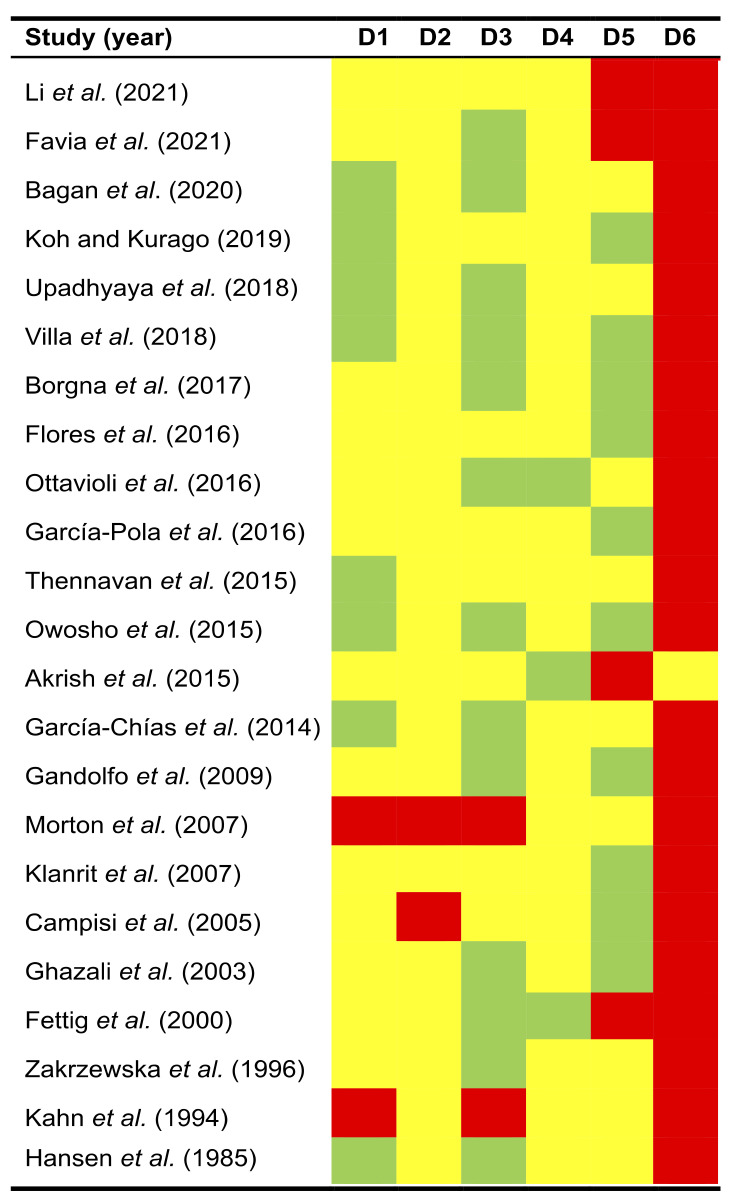
Quality plot graphically representing the risk of bias (RoB) analysis. The most relevant sources of bias were assessed in primary-level studies using the Quality in Prognosis Studies tool (QUIPS) -developed by Cochrane Prognosis Methods Group- across the following six domains: (D1) Study participation, (D2) Study attrition, (D3) Prognostic Factor Measurement, (D4) Outcome Measurement, (D5) Study confounding, and (D6) Statistical Analysis and Reporting. RoB was scored as low RoB (depicted in green colour), moderate RoB (yellow), or high RoB (red) for each domain.

The quality plot (Figure 2) depicts the RoB analysis conducted using the QUIPS tool. Potential sources of bias were classified and summarized across the following six domains:

*Study participation*. RoB was low in 34.78% of reviewed studies, moderate in 56.52%, and high in 8.70% (Figure 2). Most frequent biases were related to the lack of an adequate description of the characteristics of the sample of patients with PVL that developed oral carcinomas. In general, all studies reported the origin of the patients, and although the PVL and cancer samples were small, this is a logical aspect and difficult to resolve given the low prevalence of PVL.

*Study attrition*. RoB was moderate in 91.30% of the reviewed studies and high in 8.70% (Figure 2). The lack of reporting essential information on the follow-up period was a frequent potential source of bias. No study reported the dropout rate during the follow-up period and/or the attempt to collect information from lost patients, reasons for follow-up drop out, or the description of the characteristics of lost patients and/or those who completed the full follow-up period. This information is essential to ensure that the final sample adequately represents the baseline sample reported in primary-level studies.

*Prognostic factor measurement*. RoB was low in 56.52% of the reviewed studies, moderate in 34.78%, and high in 8.70% (Figure 2). The most relevant potential bias found was the failure to report the criteria used in primary-level studies for PVL diagnosis or the use of non-exhaustive clinical or histopathological criteria elaborated under consensus or endorsed by scientific publications.

*Outcome measurement*. RoB was low in 13.04% of reviewed studies and moderate in 86.96% (Figure 2). Mainly due to the diagnosis of oral cancer is universal and very probably not biased, the most frequent bias found was the failure to report the clinicopathological characteristics with prognostic value of these patients and their carcinomas (T status, N, M, clinical stage, etc.).

*Study confounding*. RoB was low in 43.48% of reviewed studies, moderate in 39.13%, and high in 17.39% of the reviewed studies (Figure 2). The most frequent biases found were not taking into account in the study design potentially confounding factors (e.g., age, smoking, alcohol consumption) or the failure to measure them.

*Statistical analysis and reporting*. RoB was moderate in 4.35% of reviewed studies and high in 96.65% (Figure 2). The most frequent and relevant source of potential bias was the lack of use a control group in the study design (i.e., the comparison of mortality rates in patients who developed conventional oral carcinomas without prior history of proliferative verrucous leukoplakia). A single study presented a control group but did not report hazard ratios with 95% confidence intervals, essential to assess the direction, precision, and effect size of time-to-event variables (e.g., disease-free survival or overall survival).

### 3.4. Quantitative Evaluation (Meta-Analysis)

#### 3.4.1. Quantitative Evaluation of Survival Parameters of Patients with PVL-OC

**Figure 3:**
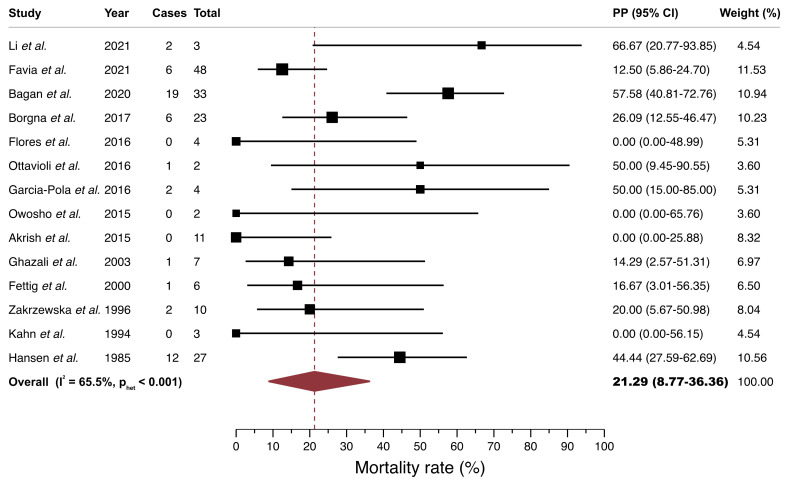
Forest plot graphically representing the meta-analysis of the mortality rate in PVL-OC. Random-effects model, DerSimonian, and Laird method. Pooled proportions (expressed as percentage) were used as effect size measure (expressed in bold). PP, pooled proportions; CI, confidence intervals; PVL-OC, oral carcinomas developed in patients with proliferative verrucous leukoplakia.

*Meta-analysis on mortality rate of patients with PVL-OC*. A random-effects model estimated a mortality pooled proportion (PP) of 21.29% (95%CI = 8.77–36.36) in patients with PVL-OC. There was a significant moderate degree of inter-study heterogeneity (*p* < 0.001, I^2^ = 65.49%; Figure 3, Table 2).

*Subgroup meta-analysis*. The meta-analysis stratified by geographical area did not show a significant variability of the mortality rate among continents (*p* = 0.06; Table 2, Figure S1, Supplementary Materials p. 6). Europe and North America maintained a mortality rate close to the overall rate (Europe: PP = 30.46%, [95%CI = 10.90–53.57]; North America: PP = 27.40% [95%CI = 6.57–53.27]), while Asia and South America obtained a much lower rate, although based on a lower sample size with imprecise and wider confidence intervals (Asia: PP = 2.78% [95%CI = 0.00–18.97]: South America: PP = 0.00% [95%CI = 0.00–48.99]).

*Meta-regression analysis*. Univariable meta-regression analyses (Table 2; Figures S2–S5, Supplementary Materials, pp. 7–10) revealed a significant lower mortality rate in patients with PVL-OC that developed verrucous carcinomas (*p* = 0.05). After performing the residual maximum likelihood (REML) method we confirmed the relevance of this variable explaining the proportion of between-study variance (adjusted R^2^ = 100%), being the most important explanatory source of heterogeneity for mortality rate in the present study. Mortality rates did not vary significantly for the rest of the potential covariates investigated (influence of follow up period [*p* = 0.44], tumor multiple development [*p* = 0.74], and conventional oral squamous cell carcinomas [*p* = 0.74]).

*Overall survival of PVL-OC (vs. conventional OSCC).* This meta-analysis could not be conducted, because only one study published sufficient data for its inclusion (i.e., presence of control group). In this study, Akrish et al. [12] reported a significantly better overall survival for patients with PVL-OC in comparison with patients with oral cancer without prior PVL (HR = 0.29 [95%CI = 0.10–0.89], *p* = 0.03; Table 2).

#### 3.4.2. Quantitative Evaluation of Clinicopathological Parameters of Patients with PVL-OC

*Meta-analysis on verrucous carcinomas in patients with PVL-OC.* A random-effects model estimated that patients with PVL frequently develop verrucous carcinomas (PP = 33.66% [95%CI = 17.58–51.43]). There was a significant high degree of inter-study heterogeneity (*p* < 0.001, I^2^ = 81.72%; Table 2, Figure S6, Supplementary Materials, p. 11).

*Meta-analysis on oral squamous cell carcinomas in patients with PVL-OC.* A random-effects model confirmed that patients with PVL mainly develop conventional oral squamous cell carcinomas (PP = 72.21% [95%CI = 52.95–88.64]). A considerable high degree of heterogeneity was found among studies (*p* < 0.001, I^2^ = 85.26%; Table 2, Figure S7, Supplementary Materials, p. 12).

*Meta-analysis on well-differentiated oral squamous cell carcinomas in patients with PVL-OC.* A random-effects model also confirmed that these patients mainly develop well-differentiated oral squamous cell carcinomas (PP = 78.41% [95%CI = 37.24–95–100.00]). A high degree of heterogeneity was observed (*p* < 0.001, I^2^ = 81.55%; Table 2, Figure S8, Supplementary Materials, p. 13).

*Meta-analysis on TNM and clinical stage in patients with PVL-OC.* A random-effects model estimated that these patients mainly develop T1/2 tumors (PP = 99.93% [95%CI = 81.37–100.0], with N- status (N+: PP = 0.60% [95%CI = 0.00–15.86]), M- status (M+: PP = 0.07% [95%CI = 0.00–18.63]), and present an early (I/II) clinical stage (PP = 89.88% [95%CI = 59.21–100.0]). Nevertheless, these meta-analyses were conducted over low sample sizes (*n* = 2 to 3 studies), showing imprecision (i.e., wide confidence intervals) and therefore, a low quality of evidence (Table 2, Figures S9–S12, Supplementary Materials, pp. 14–17).

### 3.5. Quantitative Evaluation (Secondary Analyses)

#### 3.5.1. Sensitivity Analysis

Substantial variations were not found in the overall results after the sequential repetition of meta-analyses across the sensitivity analysis series omitting one study at a time (“leave one-out” method; Tables S2–S9, Supplementary Materials, pp. 18–25). According to this secondary analysis the results of our meta-analyses do not depend on the influence of a particular individual primary-level study, reaffirming the stability of our results.

#### 3.5.2. Analysis of Small-Study Effects

Visual inspection analysis of funnel plots’ asymmetry and the Egger’s regression tests confirmed the absence of small-study effects for all parameters under investigation (mortality rate: *p*_Egger_ = 0.65; verrucous carcinomas: *p*_Egger_ = 0.77; oral squamous cell carcinomas: *p*_Egge r_ = 0.44; well-differentiated squamous cell carcinomas: *p*_Egger_ = 0.87; N status: *p*_Egger_ = 0.90; clinical stage: *p*_Egger_ = 0.87; T and M status: not applied [*n* = 2 studies, respectively]; Figure S13–S18, Supplementary Materials, pp. 26–31); therefore, biases such as publication bias could be potentially ruled out, reaffirming the reliability of our results.

## 4. Discussion

Our current systematic review carried out on a total of 23 studies and 543 patients with PVL indicates that 288 patients developed 504 oral carcinomas. Today it is accepted that PVL is the OPMD that presents the highest malignant transformation rate, close to 50% of cases [2,3] jointly with a high probability of multiple tumors development behaving as a field of cancerization [8,9,56,57]. Different therapeutic approaches have been reported for the management of PVL, including conventional surgery, CO2 laser therapy, or even pharmacological treatment, although none of them have been satisfactory and so this OPMD has also been shown to be resistant to any form of treatment with frequent recurrence of lesions on which new carcinomas may appear [1,58,59]. Despite all this, there is limited knowledge about the prognosis of carcinomas developed on PVL. Hypothetically, in a condition such as PVL, with a very high rate of malignancy and a notable tendency to the development of multiple tumors, the prognosis of carcinomas that appear during its evolution should be poor. However, our meta-analytical results demonstrate otherwise. In our study we have found an overall mortality of 21.29% for PVL-OC. Although the published series, with the exception of one paper [22], do not offer data relative to a control group, this mortality seems considerably lower than the mortality of conventional carcinoma not developed from PVL, published in previous official reports such as the SEER program (Surveillance, Epidemiology, and End Results Program-SEER [10]. This program provides information on cancer statistics among the U.S.) that reported a mortality of 34.70%, as well as in other seminal papers [11] that indicate a mortality close to 50%; in the Akrish et al. series [22], the only group that uses a control group for conventional cancers, a probability of death from PVL-OC is significantly lower (*p* = 0.03) than the probability of death from a conventional carcinoma. The reason for justifying this low mortality is currently unknown. There are not enough studies that offer data on the TNM parameters and PVL-OC stage, so we cannot provide any information in this regard. However, in relation to the influence of the PVL-OC lineage, some interesting considerations should be made. Our meta-analysis shows that 33.66% of the patients developed verrucous carcinomas while 72.21% developed OSCCs. Likewise, we verified that those series that presented higher proportions of verrucous carcinomas showed a better patient survival (*p* = 0.05), which is absolutely logical if we take into account that verrucous carcinoma exclusively develop local malignancy with no tendency to metastasis; on the contrary, in those series that presented higher proportions of OSCCs, mortality did not worsen (*p* = 0.74), which indicates in our view that PVLs generate OSCC in themselves with a good prognosis. Our group has recently reported that OSCCs developed on oral lichen planus (OLP) also present a good prognosis with low mortality rates (15.48%) [60], which we attribute to the fact that cancers developed on OLP themselves have a better behavior related with their smaller size, less tendency to generate metastasis, early stage, and good differentiation. Although there are insufficient data to extrapolate these results to PVL-OC, series reporting the degree of PVL-OC differentiation (8 studies, 49 cancer patients) indicate a majority belong to well-differentiated carcinomas (78.41%).

Our results regarding multiple tumor development in PVL are also of interest. It could be expected that the development of multiple tumors in PVL could lead to higher patient’s mortality. However, this is not the case in our meta-analysis, mortality was not affected by PVL giving rise to multiple tumors (*p* = 0.74). This observation has also been made and reported by our research group in patients who develop multiple carcinomas in OLP [60]. We think that the development of multiple tumors in OLP will not affect survival if all of them display very good prognostic parameters, and perhaps the same interpretation could be given for the multiple tumors that develop in PVL, although this is a hypothesis that should be tested. Another comment of interest concerns the influence of follow-up on mortality in the series. It could be hypothesized that, since PVL has a high malignant rate among OPMDs, with a high frequency of multiple tumors, and recurrent after treatment, long prolonged follow-up periods should be accompanied by higher mortality rates. However, this was not the case in our meta-analysis, the follow-up did not affect the survival of the series (*p* = 0.44), which is probably also indicating the good behavior of PVL-OC related on characteristics inherent to their own biopathology.

We also should point out that the studies included in this systematic review and meta-analysis have not been conducted with the same rigor, most of them presenting a high potential risk of bias. Based on our qualitative analysis, we recommend that (1) future studies investigating the behavior of oral carcinomas in PVL patients report in greater detail the demographic characteristics of patients with cancer development; (2) the follow-up periods must be long and well communicated; this is essential in prognostic studies, where it must be guaranteed a minimum period of time that allows the appearance of the investigated event (i.e., oral cancer development); (3) studies should diagnose patients with PVL based on exhaustive diagnostic criteria, preferably evidence-based (e.g., Gonzalez-Moles et al. 2021 [59]); (4) the diagnosis of cancer is universal and reliable in studies, but the clinicopathological characteristics of patients with PVL who develop cancer should be communicated in greater detail (e.g., T/N/M-status, clinical stage, etc.); (5) the analysis of potentially confounding factors (e.g., sex, age, tobacco consumption) is the Achilles heel of observational studies, a correct design, analysis, and reporting is imperative taking these factors into account; (6) only one study presented a control group and probably some of the included studies constitute sources of indirect evidence (i.e., studies not conducted or designed with the same aim of comparing the prognostic value of PVL vs. conventional carcinomas), which decreases the quality of the evidence. Therefore, a careful study design adopting an appropriate control group is essential to achieve the goals of future research papers investigating the behavior of carcinomas that develop in patients with PVL.

Our study also presents some potential limitations that should also be discussed. First, a considerable degree of inter-study heterogeneity was observed, a frequent finding in meta-analyses of proportions [61]. Consequently, random-effects models were carried out in all meta-analyses to account for heterogeneity. In addition, we performed random-effects meta-regression analyses performing the residual maximum likelihood (REML) to produce an adjusted R^2^ statistic, which estimates the proportion of the inter-study variance explained by covariates; important explanatory sources of heterogeneity were found after the application of this method (e.g., the impact and variability on mortality rates in patients with PVL which developed verrucous carcinomas and oral squamous cell carcinomas). Second, the sample sizes of the primary-level studies were very small (i.e., low number of cancers per study). However, due to the low prevalence of PVL, this limitation represents a difficult challenge to overcome. Future multicenter cohort studies should be developed to obtain more precise results, with narrower confidence intervals, providing a higher quality of evidence. Finally, an inherent limitation of the included primary-level studies -in our methodological reflections on RoB- was the failure to report relevant information and parameters, limiting the number of observations in secondary analyses (e.g., tobacco consumption or alcohol drinking). Given the methodological and clinical relevance of these variables, future studies should publish their datasets in a more rigorous way, preferably reporting individual patient data. Despite the above limitations, our systematic review and meta-analysis is innovative—showing for the first time more precise and evidence-based results, derived from-208 PVL-OC which developed 504 oral carcinomas—robust, and reliable; it is also supported by our sensitivity analyses and funnel plots and provides recommendations for the design of future studies on this topic.

## 5. Conclusions

In conclusion, our systematic review and meta-analysis presents consistent results and evidence that PVL-OC show a favorable prognostic behavior hypothetically related to characteristics inherent to the tumor’s own biopathology. Future studies on this topic should report prognostic clinical–pathological data for these carcinomas in order to elucidate the reasons for this statement.

## Figures and Tables

**Table 1:** Summarized characteristics of reviewed studies.

Total	23 Studies
Year of publication	1985–2021
Number of patients	
PVL	543
Developing oral cancer	288
Number of tumors	504
Sample size, range	
PVL	3–81
Developing oral cancer	1–48
Number of tumors	1–130
Study design	
Retrospective longitudinal	23 studies
Prospective longitudinal	1 studies
Follow up periods	
Mean of means	65.63 months
Range	14–174
Geographical region	
Europe	9 studies, 4 countries: France, Italy, Spain, UK
North America	9 studies, 1 country: USA
Asia	3 studies, 3 countries: India, Israel, Malaysia
South America	1 study, 1 country: Brazil
Multicontinent	1 study, 2 countries: Brazil-USA
Total	4 continents (9 countries)

Table S1 (Supplementary Materials, pp. 3–5) exhibits in more detail the characteristics of each study.

**Table 2:** Meta-analyses of oral cancer characteristics in patients with proliferative verrucous leukoplakia.

					Pooled Data		Heterogeneity				
Meta-Analyses	No. of Studies	No. of Patients	Stat. Model	Wt	ES (95% CI)	*p*-Value	*p_het_*	I^2^ (%)			Supplementary Materials ^a^
SURVIVAL PARAMETERS											
Mortality of patients with PVL-OC ^b^	14	183	REM	D-L	PP = 21.29% (8.77–36.36)	—	<0.001	65.49			Manuscript, Figure 3
Subgroup analysis by geographical region ^c^						0.06					Figure S1, p. 6
Europe	6	120	REM	D-L	PP = 30.46% (10.90–53.57)	—	<0.001	75.44			
North America	5	41	REM	D-L	PP = 27.40% (6.57–53.27)	—	0.22	30.97			
Asia	2	18	REM	D-L	PP = 2.78% (0.00–18.97)	—	—	—			
South America	1	4	—	—	PP = 0.00% (0.00–48.99)	—	—	—			
Univariable meta-regression ^e^											
Follow up (months, mean)	14	183	random-effects meta-regression		Coef = 0.003 (−0.005 to 0.011)	0.44 ± 0.005 ^f^	het_explained_ = 12.34% ^g^				Figure S2, p. 7
Multiple tumor development (tumors per patient, ratio)	12	150	random-effects meta-regression		Coef = −0.04 (−0.237 to 0.157)	0.74 ± 0.004 ^f^	het_explained_ = −35.94% ^g^				Figure S3, p. 8
Verrucous carcinomas (proportion of tumors, %)	12	150	random-effects meta-regression		Coef = −0.009 (−0.019 to 0.001)	0.05 ± 0.002 ^f^	het_explained_ = 100%^g^				Figure S4, p. 9
Oral squamous cell carcinomas (proportion of tumors, %)	12	150	random-effects meta-regression		Coef = 0.001 (−0.007 to 0.009)	0.74 ± 0.004 ^f^	het_explained_ = −52.34% ^g^				Figure S5, p. 10
Prognostic value of PVL-OC (vs canonical OC) ^d^											
Overall survival	1	60	—	—	HR = 0.29 (0.10–0.89)	0.03	—		—		—
**CLINICO-PATHOLOGICAL PARAMETERS**											
Verrucous carcinomas ^b^ (VC PVL-OC, %)	20	255	REM	D-L	PP = 33.66% (17.58–51.43)	—	<0.001			81.72	Figure S6, p. 11
Squamous cell carcinomas ^b^ (OSCC PVL-OC, %)	20	255	REM	D-L	PP = 72.21% (52.95–88.64)	—	<0.001			85.26	Figure S7, p. 12
Differentiation grade ^b^ (Well-differentiated SCC PVL-OC, %)	8	49	REM	D-L	PP = 78.41% (37.24–100.0)	—	<0.001			81.55	Figure S8, p. 13
T status ^b^ (T1/2 PVL-OC, %)	2	13	REM	D-L	PP = 99.93% (81.37–100.0)	—	—			—	Figure S9, p. 14
N status ^b^ (Lymph node(+) PVL-OC, %)	3	19	REM	D-L	PP = 0.60% (0.00–15.86)	—	0.40			0.00	Figure S10, p. 15
M status ^b^ (Distance Metastasis(+) PVL-OC, %)	2	13	REM	D-L	PP = 0.07% (0.00–18.63)	—	—			—	Figure S11, p. 16
Clinical stage ^b^ (Early stage (I/II) PVL-OC, %)	3	46	REM	D-L	PP = 89.88% (59.21–100.0)	—	0.07			63.03	Figure S12, p. 17

Abbreviations: Stat., statistical; Wt, method of weighting; ES, effect size estimation; PP, pooled proportion; HR, hazard ratio; CI, confidence intervals; REM, random-effects model; D-L, DerSimonian and Laird method, PVL, proliferative verrucous leukoplakia; OC, oral carcinoma, PVL-OC, oral carcinomas arising in patients with pre-existing proliferative verrucous leukoplakia; SCC, squamous cell carcinoma. a—More information in the supple, b—Proportion meta-analysis, c—Proportion meta-analyses (Subgroup analyses), d—Prognosis meta-analysis, e—Effect of study covariates on the mortality rate among patients with PVL-OC. A meta-regression coefficient >0 indicates a greater impact of covariates on the mortality rate. f—*p*-value ± standard error recalculated after 10,000 permutations based on Montecarlo simulations, g—Proportion of between-study variance explained (adjusted R^2^ statistic) using the residual maximum likelihood (REML) method. A negative number for proportion of heterogeneity explained reflects no heterogeneity explained.

## Data Availability

The data that supports the findings of this study are available in the Supplementary Material of this article.

## Supplementary Materials

The following are available online at https://www.mdpi.com/article/10.3390/cancers13194843/s1, Table S1. Characteristics of the included studies. Table S2. Sensitivity analysis of studies included in the meta-analysis on mortality rate among patients with PVL-OC. Table S3. Sensitivity analysis of studies included in the meta-analysis on proportion of verrucous carcinomas among patients with PVL-OC. Table S4. Sensitivity analysis of studies included in the meta-analysis on proportion of oral squamous cell carcinomas among patients with PVL-OC. Table S5. Sensitivity analysis of studies included in the meta-analysis on proportion of well-differentiated oral squamous cell carcinomas among patients with PVL-OC. Table S6. Sensitivity analysis of studies included in the meta-analysis on proportion of T1/2 oral carcinomas among patients with PVL-OC. Table S7. Sensitivity analysis of studies included in the meta-analysis on proportion of N+ status among patients with PVL-OC. Table S8. Sensitivity analysis of studies included in the meta-analysis on proportion of M+ status among patients with PVL-OC. Table S9. Sensitivity analysis of studies included in the meta-analysis on proportion of N+ status among patients with PVL-OC. Figure S1. Forest plot graphically representing the subgroup meta-analysis of the mortality rate of patients with PVL-OC, stratified by geographical area, Figure S2. Bubble plot graphically representing the potential effect of mean follow up period (expressed in months) on the mortality rate. Figure S3. Bubble plot graphically representing the potential effect of multiple tumour development (estimated as a tumours per patient ratio) on the mortality rate. Figure S4. Bubble plot graphically representing the potential effect of verrucous carcinomas (expressed as % of tumours) on the mortality rate. Figure S5. Bubble plot graphically representing the potential effect of oral squamous cell carcinomas (expressed as % of tumours) on the mortality rate. Figure S6. Forest plot graphically representing the meta-analysis of verrucous carcinomas in patients with PVL-OC (proportion of patients with verrucous carcinomas). Figure S7. Forest plot graphically representing the meta-analysis of oral squamous cell carcinomas in patients with PVL-OC (proportion of patients with oral squamous cell carcinomas). Figure S8. Forest plot graphically representing the meta-analysis of well differentiated OSCCs in patients with PVL-OC (proportion of patients with well differentiated OSCCs). Figure S9. Forest plot graphically representing the meta-analysis of T status parameter in patients with PVL-OC (proportion of patients with T1/2). Figure S10. Forest plot graphically representing the meta-analysis of N status parameter in patients with PVL-OC (proportion of patients with N+ status). Figure S11. Forest plot graphically representing the meta-analysis of M status parameter in patients with PVL-OC (proportion of patients with M+ status). Figure S12. Forest plot graphically representing the meta-analysis of clinical stage parameter in patients with PVL-OC (proportion of patients with I/II stage). Figure S13. A funnel plot of estimated transformed proportions against their standard errors, graphically representing the analysis of small-study effects on the mortality rate parameter in patients with PVL-OC. Figure S14. A funnel plot of estimated transformed proportions against their standard errors, graphically representing the analysis of small-study effects on the proportion of verrucous carcinomas in patients with PVL-OC. Figure S15. A funnel plot of estimated transformed proportions against their standard errors, graphically representing the analysis of small-study effects on the proportion of oral squamous cell carcinomas in patients with PVL-OC. Figure S16. A funnel plot of estimated transformed proportions against their standard errors, graphically representing the analysis of small-study effects on the proportion of well-differentiated oral squamous cell carcinomas in patients with PVL-OC. Figure S17. A funnel plot of estimated transformed proportions against their standard errors, graphically representing the analysis of small-study effects on the proportion of N+ status cases among patients with PVL-OC. Figure S18. A funnel plot of estimated transformed proportions against their standard errors, graphically representing the analysis of small-study effects on the proportion of I/II-stage cases among patients with PVL-OC. List S1. List of excluded studies with reasons.
