# Supplementary material for: Prognosis Parameters of Oral Carcinomas Developed in Proliferative Verrucous Leukoplakia: A Systematic Review and Meta-Analysis
Source: Cancers (Basel). 2021 Sep 28;13(19):4843. doi: 10.3390/cancers13194843 (PMC8507842; doi:10.3390/cancers13194843)
Supplement: Supplementary file 1 [file cancers-13-04843-s001.zip › cancers-1386404-supplementary.pdf]

Supplementary Materials

PROGNOSIS PARAMETERS OF ORAL CARCINOMAS DEVELOPED IN PROLIFERATIVE VERRUCOUS LEUKOPLAKIA: A SYSTEMATIC REVIEW AND META-ANALYSIS.

Miguel Ángel González-Moles[a,b], Saman Warnakulasuriya[c,d], Pablo Ramos-García[a,b]

[a] - School of Dentistry, University of Granada, Granada, Spain.

[b] - Biohealth Research Institute, Granada, Spain.

[c] - Oral Medicine Department, King's College London, London, UK.

[d] - WHO Collaborating Centre for Oral Cancer, London, UK.

Table of contents

1. Characteristics of the included studies 3

2. Subgroup meta-analysis on mortality rate of patients with PVL-OC, stratified by geographical area 6

3. Meta-regression analyses 7

- Effect of follow-up period on the mortality rate of patients with PVL-OC 7
- Effect of multiple tumor development 8
- Effect of verrucous carcinomas 9
- Effect of oral squamous cell carcinomas 10

4. Meta-analysis on verrucous carcinomas in patients with PVL-OC 11

5. Meta-analysis on squamous cell carcinomas in patients with PVL-OC 12

6. Meta-analysis on well-differentiated SCCs in patients with PVL-OC 13

7. Meta-analysis on T status in patients with PVL-OC 14

8. Meta-analysis on N status in patients with PVL-OC 15

9. Meta-analysis on M status in patients with PVL-OC 16

10. Meta-analysis on clinical stage in patients with PVL-OC 17

11. Sensitivity analyses (leave-one-out method) of meta-analyses on survival and clinic-pathological parameters in patients with PVL-OC

- Mortality rate 18
- Verrucous carcinomas 19
- Oral squamous cell carcinomas 20
- Well-differentiated squamous cell carcinomas 21
- T status 22
- N status 23
- M status 24
- Clinical stage 25

12. Analysis of small-study effects: Funnel plots

- Mortality rate 26
- Verrucous carcinomas 27
- Oral squamous cell carcinomas 28
- Well-differentiated squamous cell carcinomas 29
- N status 30
- Clinical stage 31

13. List of excluded studies with reasons 32

Table S1 – Characteristics of the included studies (n=23)

Author (year)	Country	Study design (recruitment period)	Diagnostic criteria for PVL	Follow up, m	Patients with proliferative verrucous leukoplakia				Oral carcinomas development in patients with proliferative verrucous leukoplakia								
					Sample Size, n	sex; age distribution, y: mean±SD(range)	Tobacco Smoking, n (%)	Alcohol drinking n (%)	Malignant transformation (%)	Patients, n (histopathology)	Tumours, n (histopathology)	Mortality rate, %(95%CI)*	Well-differentiated % (95%CI)*	T1/2-status, %(95%CI)*	N+ status, %(95%CI)*	M+ status, %(95%CI)*	I/II-clinical stage, %(95%CI)*
Li *et al.* (2021)	USA	Retrospective cohort (NR)	Own	Mean= 114	4	M=2 (50%)<br>F=2 (50%)<br><br>Mean age: 58±21.74	NR	NR	75%	3 (3 OSCCs)	7 (7 OSCCs)	66.67 (20.77-93.85)	NR (NR)	NR (NR)	NR (NR)	NR (NR)	NR (NR)
Favia *et al.* (2021)	Italy	Retrospective cohort (1989-2008)	Hansen *et al* (1985)	Mean= 62.45 Range = 18–240	75	NR; NR	Yes=11 No=64 (84.33%)	NR	64%	48 (15 OSCCs; 35 VCs)	130 (73 OSCCs; 57 VCs)	12.50 (5.86-24.70)	100.0 (79.61-100.0)	NR (NR)	NR (NR)	NR (NR)	NR (NR)
Bagan *et al.* (2020)	Spain	Retrospective cohort (1996-2018)	Villa *et al.*(2018)	Mean= 65.61± 77.45 Range: 12-256.8	81	M=29 (35.8%)<br>F=52 (64.2%)<br><br>Mean age: 62.6±12.3	NR	NR	40.74%	33 (33 OSCCs)	105 (105 OSCCs)	57.58 (40.81-72.76)	NR (NR)	NR (NR)	NR (NR)	NR (NR)	78.79 (62.25-89.32)
Koh and Kurago (2019)	USA	Retrospective cohort (NR)	Own	Mean= 39.6 Range = 12-84	10	M=5 (50%)<br>F=5 (50%)<br><br>Mean age: 60.7±11.94	Yes=6 No=2 (20%) Missing=2	NR	50%	5 (3 OSCCs; 3 VCs)	NR (NR)	NR (NR)	NR (NR)	NR (NR)	NR (NR)	NR (NR)	NR (NR)
Upadhyaya *et al.* (2018)	USA	Retrospective cohort (1994-2016)	Hansen *et al.*(1985)	Mean= 91.8	20	M=6 (30%)<br>F=14 (70%)<br><br>Mean age: 62.7 (range:34-87)	Yes=12 (60%) No=5 (25%) missing=3	NR	45%	9 (2 OSCCs; 6 VC; 1 Papillary)	NR (NR)	NR (NR)	NR (NR)	NR (NR)	NR (NR)	NR (NR)	NR (NR)
Villa *et al.* (2018)	USA and Brazil	Retrospective cohort (1996-2016)	Villa et al. (2018)	Mean= 47.06± 47.33	42	M=7 (16.7%)<br>F=35 (83.3%)<br><br>Mean age: 67.23±11.95	Yes=5 Former=12 Never=24 (57.14%)	Yes=13 Former= 1 Never=2 8 (66.67%)	71.43%	30 (25 OSCCs; 5 VCs)	NR (NR)	NR (NR)	NR (NR)	NR (NR)	NR (NR)	NR (NR)	NR (NR)
Borgna *et al.* (2017)	UK	Retrospective cohort (1990-2015)	Hansen *et al.*(1985)	Mean= 51.6±4 4.4	48	M=24 (50%)<br>F=24 (50%)<br><br>Mean age: 70±13	Yes=33 No=15 (31.25%)	Yes=27 No=21 (43.75%)	47.92%	23 (9 OSCCs; 11 VCs)	NR (NR)	26.09 (12.55-46.47)	100.0 (70.09-100.0)	NR (NR)	NR (NR)	NR (NR)	NR (NR)

Flores *et al.* (2016)	Brazil	Retrospective cohort (NA)	Own	Mean= 65.6±6 3.15	15	M=0 (0%) F=15 (100%) Mean age: 68.13±9.82	Yes=0 (0%) No=15 (100%)	Yes=0 No=15 (100%)	26.67%	4 (4 OSCCs; 1 VC)	6 (5 OSCCs; 1 VC)	0.00 (0.00-48.99)	NR (NR)	NR (NR)	NR (NR)	NR (NR)	NR (NR)
Ottavioli *et al.* (2016)	France	Retrospective cohort (NA)	Carrard *et al.*(2013)	Mean= 24±12	3	M=0 (0%) F=3 (100%) Mean age: 80.7±4.9	Yes=0 (0%) No=3 (100%)	NR	66.67%	2 (1 OSCC; 1 VC)	2 (1 OSCC; 1 VC)	50.00 (9.45-90.55)	100.0 (20.65-100.0)	50.00 (9.45-90.55)	0.00 (0.00-65.76)	50.00 (9.45-90.55)	50.00 (9.45-90.55)
García-Pola *et al.* (2016)	Spain	Prospective cohort (1984-2015)	Own	Mean= 174	14	M=3 (21.4%) F=11 (78.6%) Mean age: 56.4 (range: 35-69)	Former=3 No=11 (78.57%)	Yes=3 No=11 (78.6%)	28.57%	4 (3 OSCCs, 1 VC)	12 (10 OSCCs, 2 VCs)	50.00 (15.00-85.00)	100.0 (43.85-100.0)	NR (NR)	NR (NR)	NR (NR)	NR (NR)
Thennavan *et al.* (2015)	India	Retrospective cohort (NR)	Own	14	7	M=1 (14.3%) F=6 (85.7%) Mean age: 63.7 (range: 54-76)	Yes=3 No=4 (57.14%)	NR	14.29%	1 (1 OSCC)	1 (1 OSCC)	NR (NR)	NR (NR)	NR (NR)	NR (NR)	NR (NR)	NR (NR)
Owosho *et al.* (2015)	USA	Retrospective cohort (2007-2013)	Cerero-Lapiedra et al. (2010)	Mean= 56.4	7	M=4 (57.1%) F=3 (42.9%): Mean age=63.7 (range: 47-82)	Yes=0 No=7 (100%)	Yes=2 No=5 (71.43%)	28.57%	2 (1 OSCC, 1 VC)	12 (10 OSCCs, 2 VCs)	0.00 (0.00-65.76)	NR (NR)	NR (NR)	NR (NR)	NR (NR)	NR (NR)
Akrish *et al.* (2015)	Israel	Retrospective cohort (1990-2012)	Own	>70	NR	M=6 (55.5%) F=5 (45.5%) Mean age: 64	Yes=1 No=10 (90.91%)	NR	NR	11 (11 OSCCs, 5 VCs)	44 (38 OSCCs, 6 VCs)	0.00 (0.00-25.88)	NR (NR)	100.0 (74.12-100.0)	0.00 (0.00-25.88)	0.00 (0.00, 25.88)	100.0 (74.12-100.0)
García-Chías *et al.* (2014)	Spain	Retrospective cohort (1984-2011)	Cerero-Lapiedra *et al.* (2010)	Mean= 44	40	M=15 (37.5%) F=25 (62.5%) Mean age: 62.3	Yes=12 No=28 (70%)	NR	17.5%	7 (4 OSCCs, 3 VCs)	7 (4 OSCCs, 3 VCs)	NR (NR)	NR (NR)	NR (NR)	NR (NR)	NR (NR)	NR (NR)
Gandolfo *et al.* (2009)	Italy	Retrospective cohort (1981-2006)	Hansen *et al.*(1985)	Mean= 82.68	47	M=10 (21.3%) F=37 (78.7%): Age: 65.9±11.0	Yes=17 No=20 (62.8%)	Yes=12 No=35 (74.47%)	44.19%	19 (NR)	41 (32 OSCCs, 9 VCs)	NR (NR)	NR (NR)	NR (NR)	NR (NR)	NR (NR)	NR (NR)
Morton *et al.* (2007)	USA	Retrospective cohort (NR)	NR	NR	3	M=1 (33.33%) F=2 (66.67%) Age: 80±8.19	Yes=1 No=2 (66.67%)	NR	100%	3 (2 OSCCs, 1 VC)	3 (2 OSCCs, 1 VC)	NR (NR)	66.67 (20.77-93.85)	NR (NR)	NR (NR)	NR (NR)	NR (NR)

Klanrit *et al.* (2007);	USA	Retrospective cohort (1990-1999)	Own	Mean= 116	6	M=1 (16.67%) F=5 (83.33%) Age: 65.83±10.11	Yes=1 Former=1 No=3 (50%) Missing=1	Yes=1 Former= 1 No=3 (50%) Missing= 1	100%	6 (5 OSCCs, 2 VCs, 2 cuniculatum)	13 (8 OSCCs, 2 VCs, 3 cuniculatum)	NR (NR)	60.00 (23.07-88.24)	NR (NR)	NR (NR)	NR (NR)	NR (NR)
Campisi *et al.* (2004)	Italy	Retrospective cohort (NR)	Own	NR	58	M=22 (37.93%) F=36 (62.07%) Age: 66.5±12.92	Yes=8 Former=9 No=41 (70.69%)	Yes=10 No=48 (82.8%)	43.10%	25 (22 OSCCs, 3 VCs)	25 (22 OSCCs, 3 VCs)	NR (NR)	NR (NR)	NR (NR)	NR (NR)	NR (NR)	NR (NR)
Ghazali *et al.* (2003)	Malaysia	Retrospective cohort (NR)	Hansen *et al.* (1985)	Mean= 56.4	9	M=2 (22.22%) F=7 (77.78%) Mean age: 61.67±15.16	Yes=4 No=5 (55.56%)	Yes=1 No=8 (11.1%)	77.78%	7 (6 OSCCs, 4 VCs)	13 (8 OSCCs, 5 VCs)	14.29 (2.57-51.31)	NR (NR)	NR (NR)	NR (NR)	NR (NR)	NR (NR)
Fettig *et al.* (2000)	USA	Retrospective cohort (1994-1999)	Hansen *et al.* (1985)	Mean= 52.8	10	M=6, 60% F=4, 40.0% Mean age=65 (range: 51-82)	Yes=3 No=5 (50%) missing=2	NR	60.00%	6 (3 OSCCs, 2 VCs, 1 papillary)	8 (4 OSCCs, 3 VCs, 1 papillary)	16.67 (3.01-56.35)	33.33 (6.15-79.23)	NR (NR)	16.67 (3.01-56.35)	NR (NR)	NR (NR)
Zakrzewska *et al.* (1996)	UK	Retrospective cohort (NA)	Hansen *et al.* (1985)	Mean= 79.2	10	M=5 (50%) F=5 (50%) Mean age (63.5, range: 42-81)	Yes=7 No=3 (30%)	NR	100%	10 (NR)	NR (NR)	20.00 (5.67-50.98)	10.00 (1.79-40.42)	NR (NR)	NR (NR)	NR (NR)	NR (NR)
Kahn *et al.* (1994)	USA	Retrospective cohort (1988-1990)	NR	Mean= 48 Range = 24-60	4	M=2 (50%) F=2 (50%) Mean age=68.75 (range: 51-75)	Yes=2 No=2 (50%)	NR	75%	3 (3 OSCCs, 2 VCs)	6 (4 OSCCs, 2 VCs)	0.00 (0.00-56.15)	NR (NR)	NR (NR)	NR (NR)	NR (NR)	NR (NR)
Hansen *et al.*(1985)	USA	Retrospective cohort (1961-1983)	Hansen *et al.* (1985)	Mean= 73.2	30	M=6 (20%) F=24 (80%) Mean age=49 (range: 27-74)	Yes=18 No=12 (40%)	NR	90%	27 (5 OSCCs, 4 VCs, 18 papillary)	27 (5 OSCCs, 4 VCs, 18 papillary)	44.44 (27.59-62.69)	NR (NR)	NR (NR)	NR (NR)	NR (NR)	NR (NR)

*Freeman-Tukey double arcsine transformed proportions -expressed as percentages- with their corresponding Wilson’s score 95% confidence intervals.

Abbreviations: n, number; m, months; y, years; SD, standard deviation; NR, not reported; PVL, proliferative verrucous leukoplakia; OSCC, oral squamous cell carcinoma; VC, verrucous carcinoma.

2. Subgroup meta-analysis on mortality rate of patients with PVL-OC, by geographical area

Figure S1. Forest plot graphically representing the subgroup meta-analysis of the mortality rate of patients with PVL-OC, stratified by geographical area. Random-effects model, DerSimonian and Laird method.

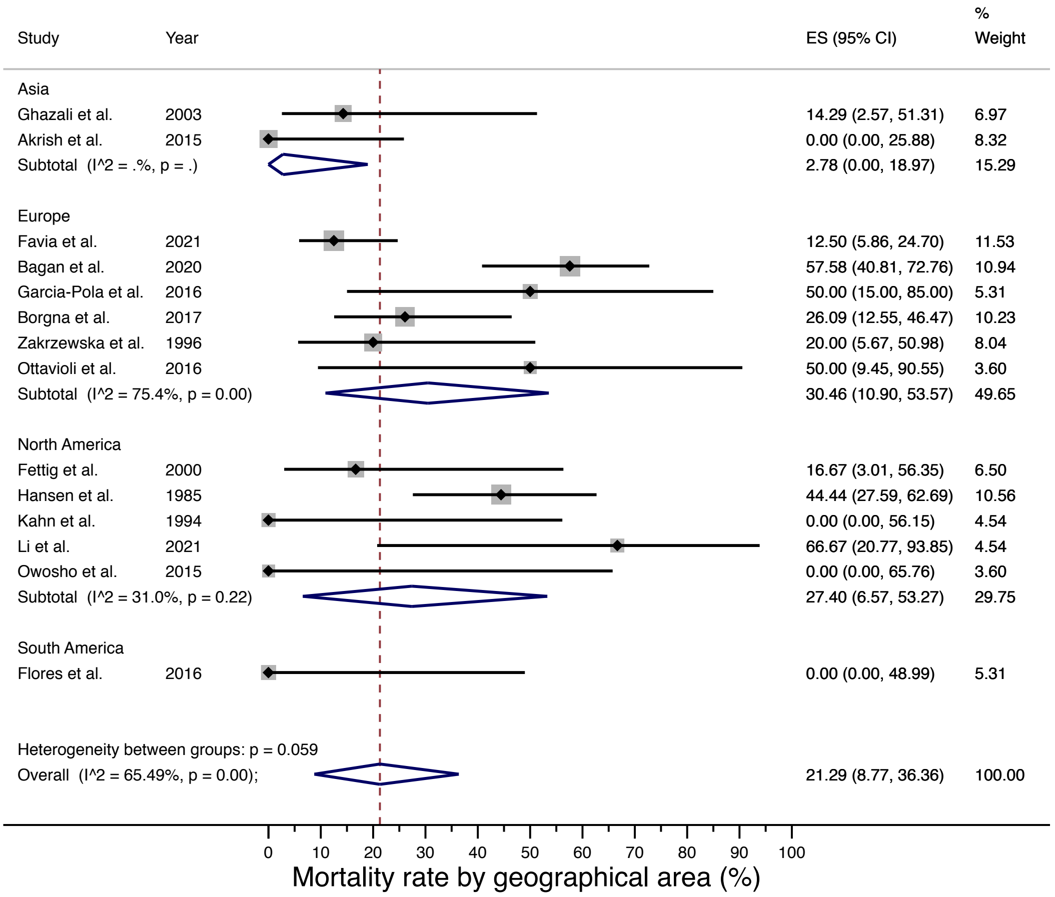

Abbreviations: ES, effect size (measured using pooled proportions); CI, confidence interval; PVL-OC, oral carcinomas developed in patients with proliferative verrucous leukoplakia.

3. Meta-regression analyses.

3.1 Effect of follow up on the mortality rate among patients with PVL-OC

Figure S2. Bubble plot graphically representing the potential effect of mean follow up period (expressed in months) on the mortality rate. The red line exhibits the fitted meta-regression line together with blue circles representing the estimates from each individual study, sized according to the precision of each estimate (the inverse of its within-study variance).

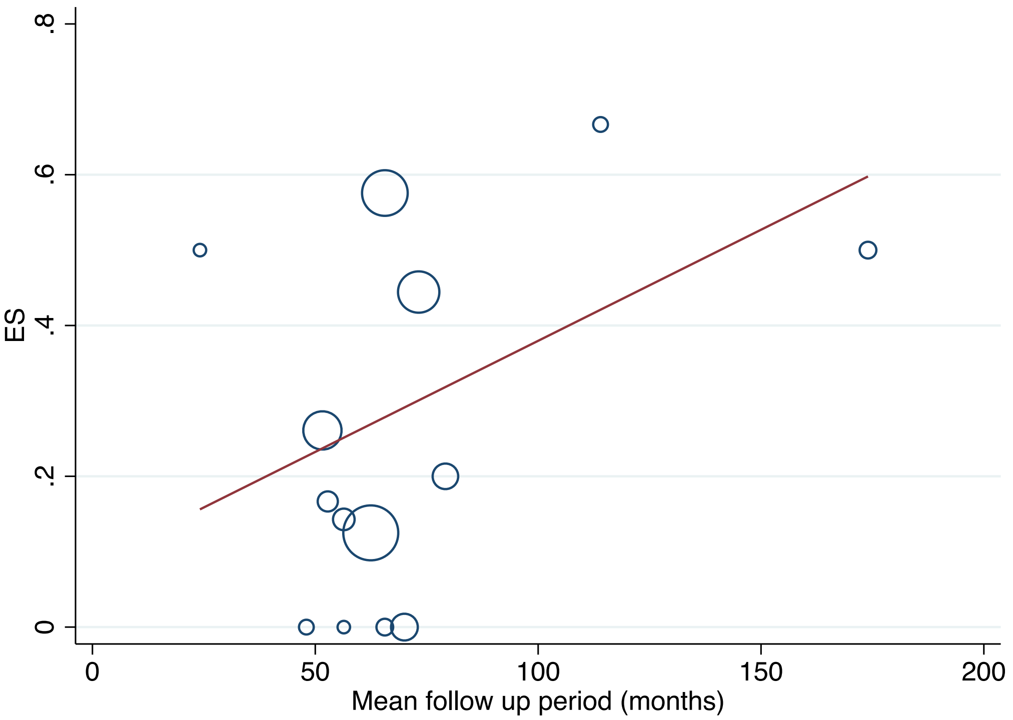

Abbreviations: ES, effect size (measured using pooled proportions); PVL-OC, oral carcinomas developed in patients with proliferative verrucous leukoplakia.

3.2 Effect of multiple tumour development on the mortality rate among patients with PVL-OC

Figure S3. Bubble plot graphically representing the potential effect of multiple tumour development (estimated as a tumours per patient ratio) on the mortality rate. The red line exhibits the fitted meta-regression line together with blue circles representing the estimates from each individual study, sized according to the precision of each estimate (the inverse of its within-study variance).

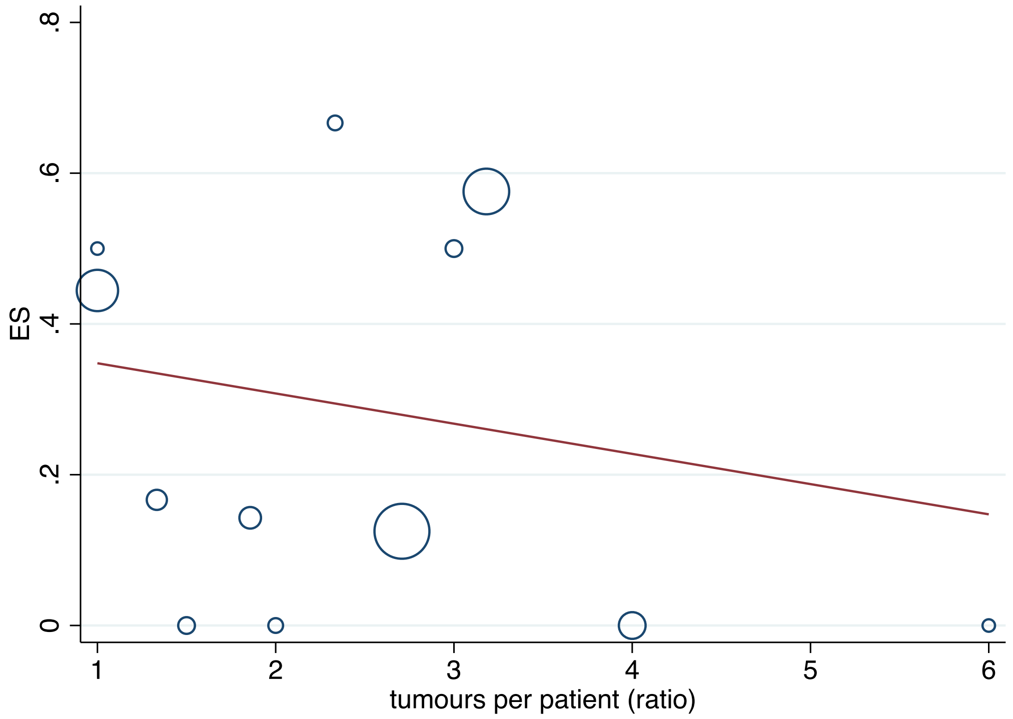

Abbreviations: ES, effect size (measured using pooled proportions); PVL-OC, oral carcinomas developed in patients with proliferative verrucous leukoplakia.

3.3 Effect of verrucous carcinomas on the mortality rate among patients with PVL-OC

Figure S4. Bubble plot graphically representing the potential effect of verrucous carcinomas (expressed as % of tumours) on the mortality rate. The red line exhibits the fitted meta-regression line together with blue circles representing the estimates from each individual study, sized according to the precision of each estimate (the inverse of its within-study variance).

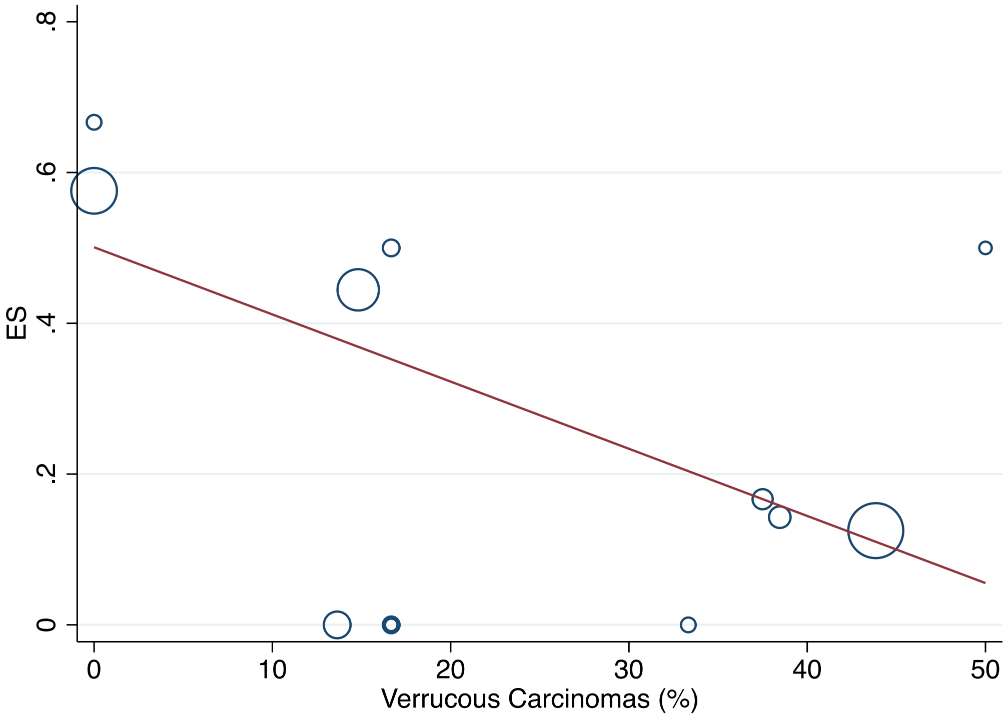

Abbreviations: ES, effect size (measured using pooled proportions); PVL-OC, oral carcinomas developed in patients with proliferative verrucous leukoplakia.

3.4 Effect of oral squamous cell carcinomas on the mortality rate among patients with PVL-OC

Figure S5. Bubble plot graphically representing the potential effect of oral squamous cell carcinomas (expressed as % of tumours) on the mortality rate. The red line exhibits the fitted meta-regression line together with blue circles representing the estimates from each individual study, sized according to the precision of each estimate (the inverse of its within-study variance).

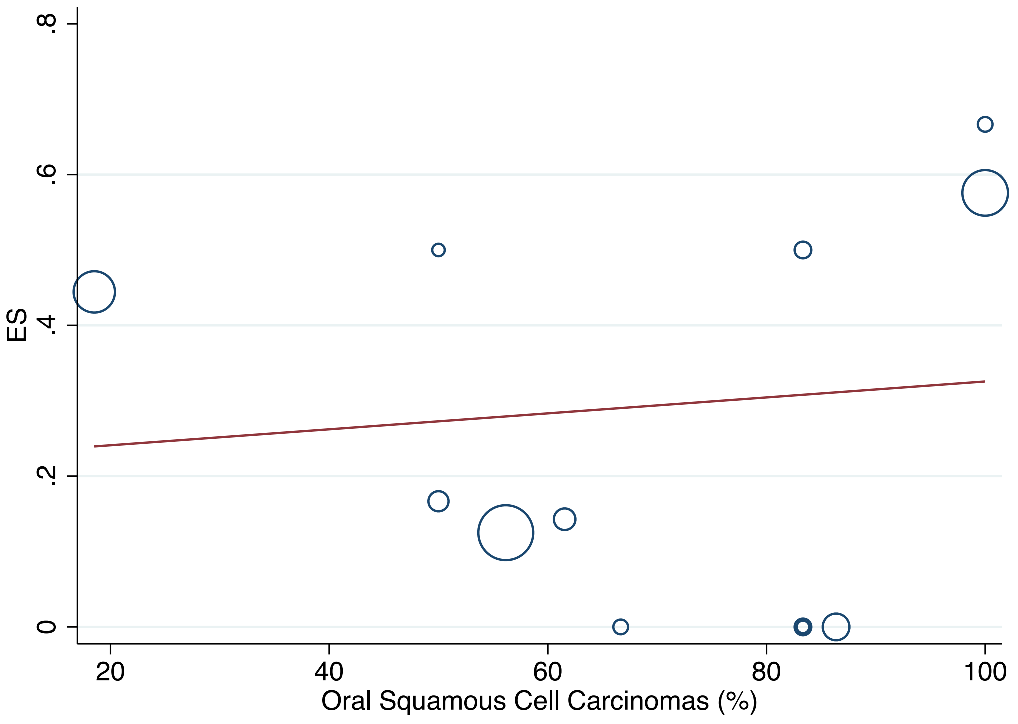

Abbreviations: ES, effect size (measured using pooled proportions); PVL-OC, oral carcinomas developed in patients with proliferative verrucous leukoplakia.

4. Meta-analysis on verrucous carcinomas in patients with PVL-OC

Figure S6. Forest plot graphically representing the meta-analysis of verrucous carcinomas in patients with PVL-OC (proportion of patients with verrucous carcinomas). Random-effects model, DerSimonian and Laird method.

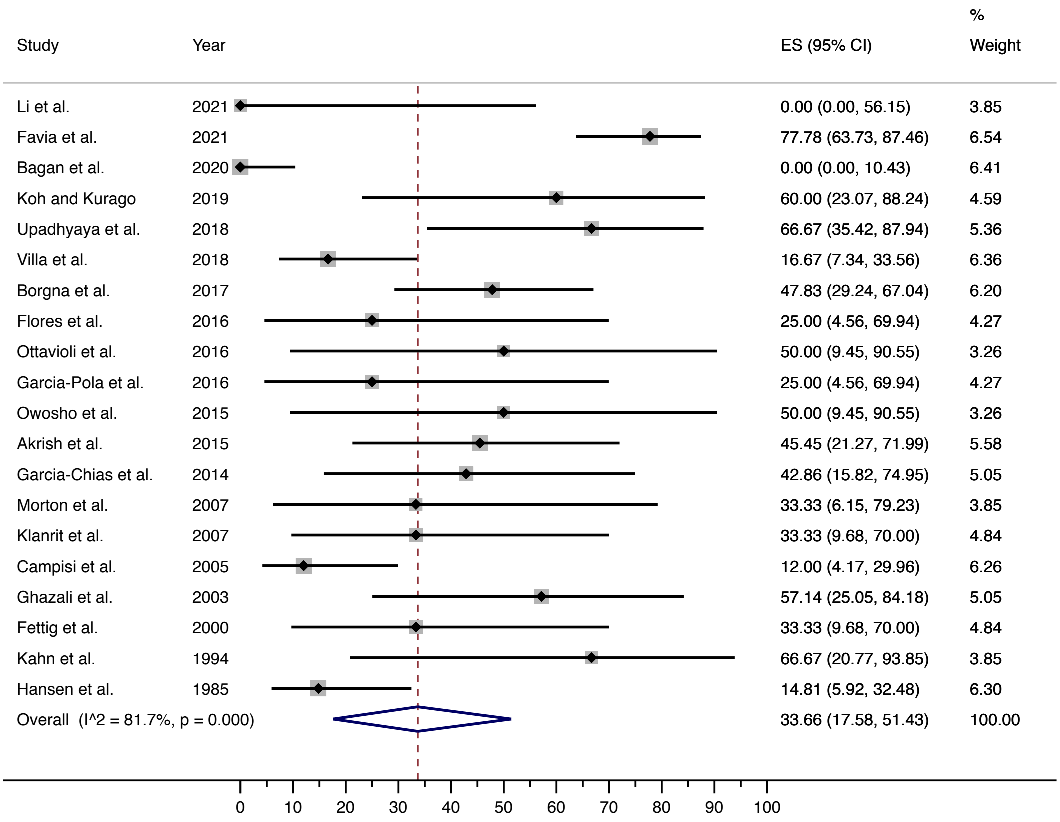

Abbreviations: ES, effect size (measured using pooled proportions); CI, confidence interval; PVL-OC, oral carcinomas developed in patients with proliferative verrucous leukoplakia.

5. Meta-analysis on squamous cell carcinomas in patients with PVL-OC

Figure S7. Forest plot graphically representing the meta-analysis of oral squamous cell carcinomas in patients with PVL-OC (proportion of patients with oral squamous cell carcinomas). Random-effects model, DerSimonian and Laird method.

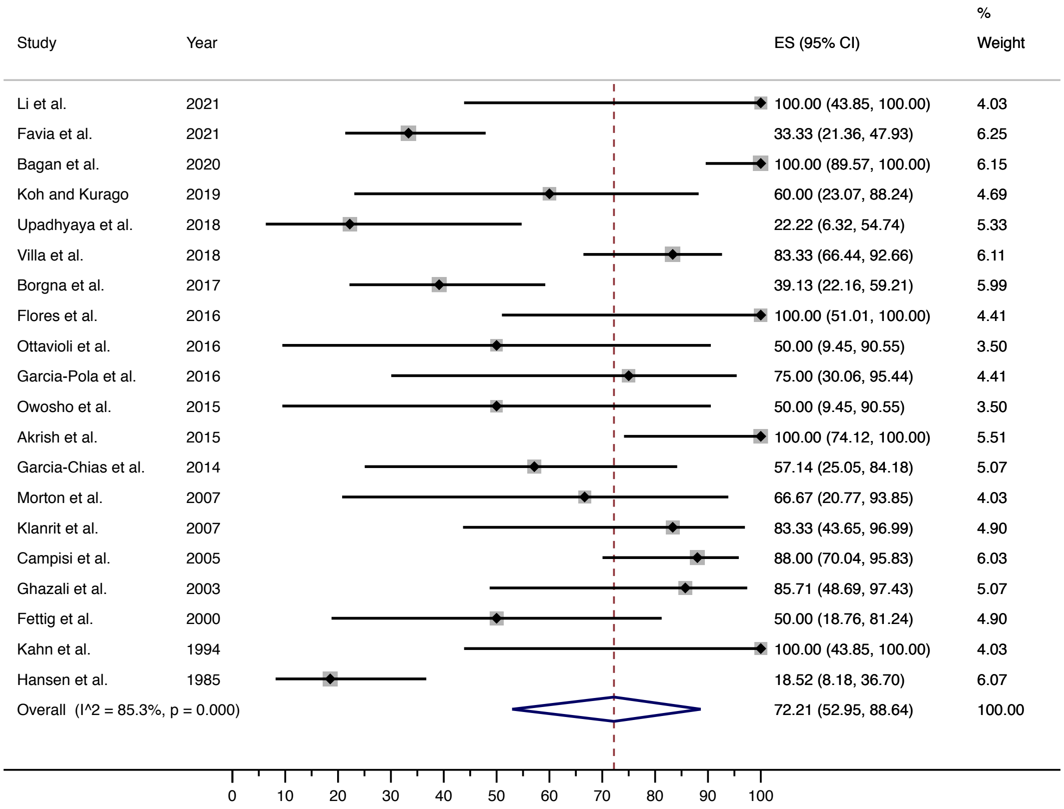

Abbreviations: ES, effect size (measured using pooled proportions); CI, confidence interval; PVL-OC, oral carcinomas developed in patients with proliferative verrucous leukoplakia.

6. Meta-analysis on well-differentiated SCCs in patients with PVL-OC

Figure S8. Forest plot graphically representing the meta-analysis of well differentiated OSCCs in patients with PVL-OC (proportion of patients with well differentiated OSCCs). Random-effects model, DerSimonian and Laird method.

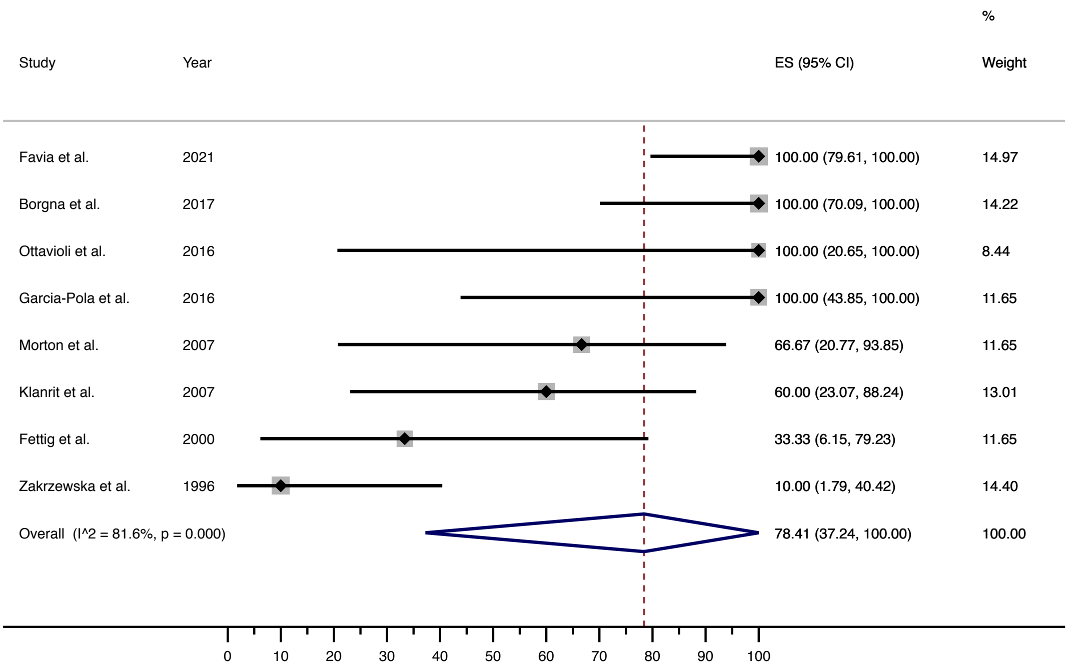

Abbreviations: ES, effect size (measured using pooled proportions); CI, confidence interval; PVL-OC, oral carcinomas developed in patients with proliferative verrucous leukoplakia; OSCC, oral squamous cell carcinomas.

7. Meta-analysis on T status in patients with PVL-OC

Figure S9. Forest plot graphically representing the meta-analysis of T status parameter in patients with PVL-OC (proportion of patients with T1/2). Random-effects model, DerSimonian and Laird method.

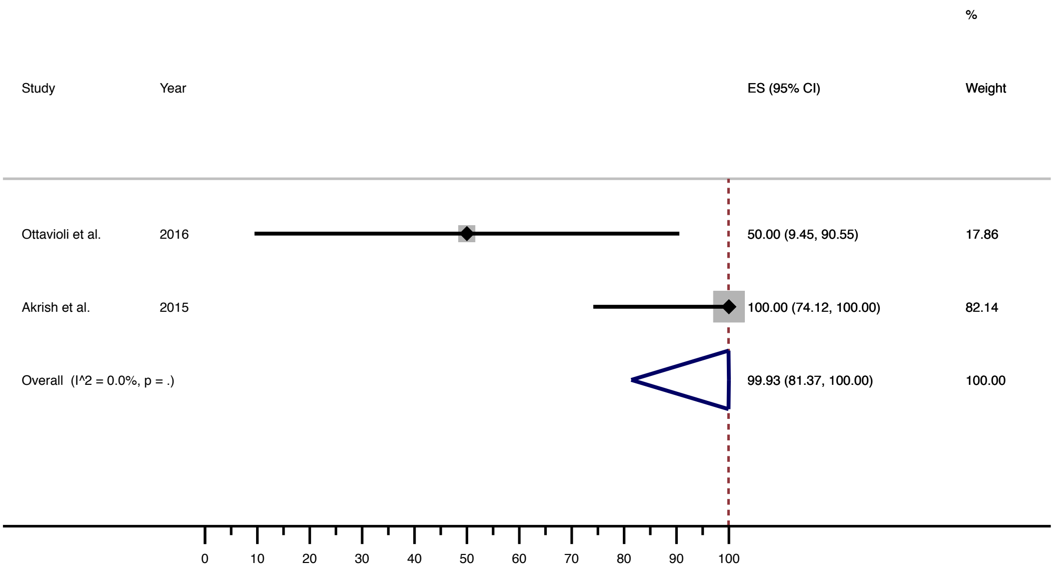

Abbreviations: ES, effect size (measured using pooled proportions); CI, confidence interval; PVL-OC, oral carcinomas developed in patients with proliferative verrucous leukoplakia.

8. Meta-analysis on N status in patients with PVL-OC

Figure S10. Forest plot graphically representing the meta-analysis of N status parameter in patients with PVL-OC (proportion of patients with N+ status). Random-effects model, DerSimonian and Laird method.

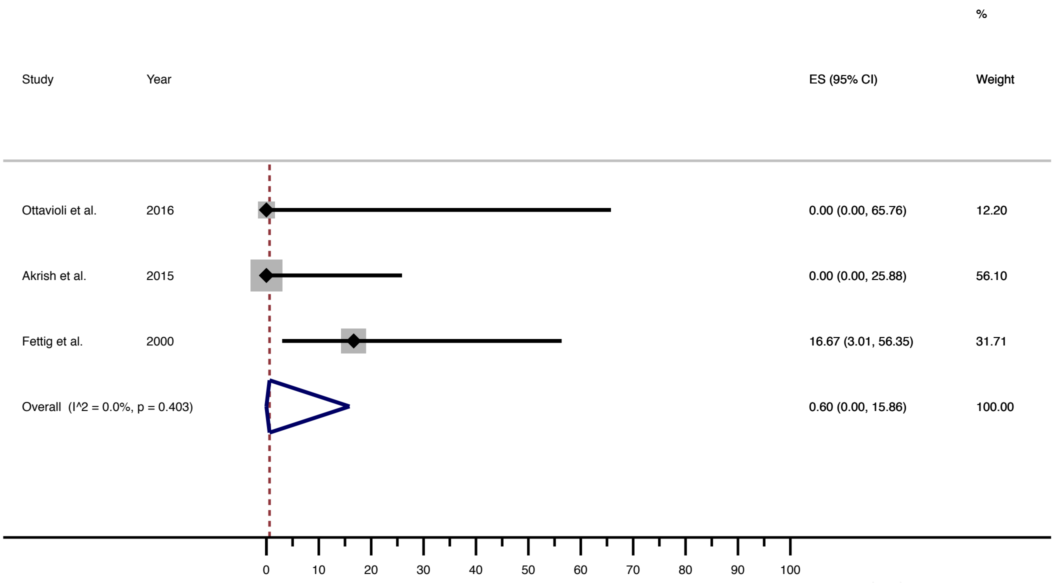

Abbreviations: ES, effect size (measured using pooled proportions); CI, confidence interval; PVL-OC, oral carcinomas developed in patients with proliferative verrucous leukoplakia.

9. Meta-analysis on M+ status in patients with PVL-OC

Figure S11. Forest plot graphically representing the meta-analysis of M status parameter in patients with PVL-OC (proportion of patients with M+ status). Random-effects model, DerSimonian and Laird method.

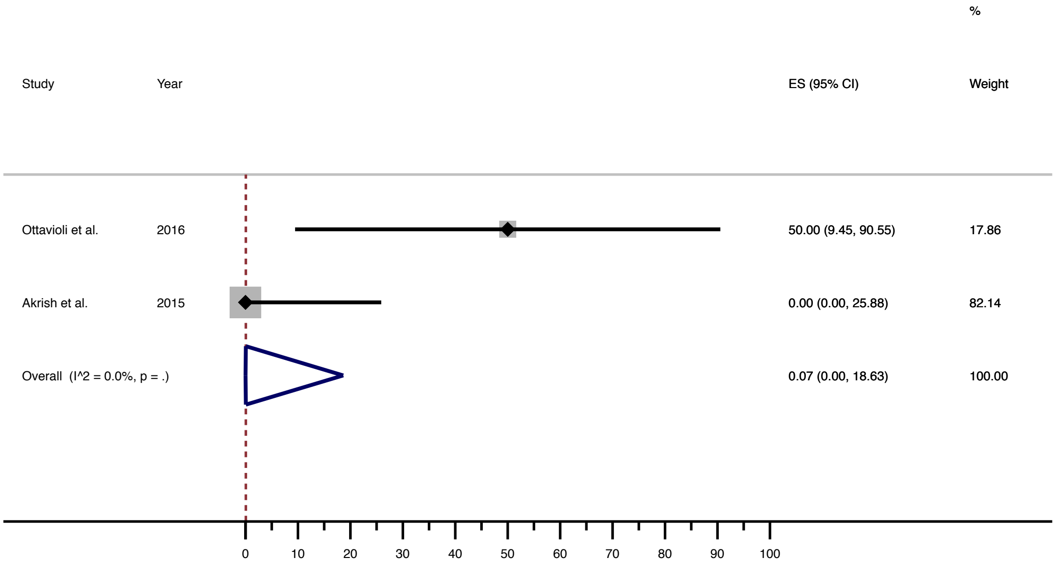

Abbreviations: ES, effect size (measured using pooled proportions); CI, confidence interval; PVL-OC, oral carcinomas developed in patients with proliferative verrucous leukoplakia.

10. Meta-analysis on clinical stage in patients with PVL-OC

Figure S12. Forest plot graphically representing the meta-analysis of clinical stage parameter in patients with PVL-OC (proportion of patients with I/II stage). Random-effects model, DerSimonian and Laird method.

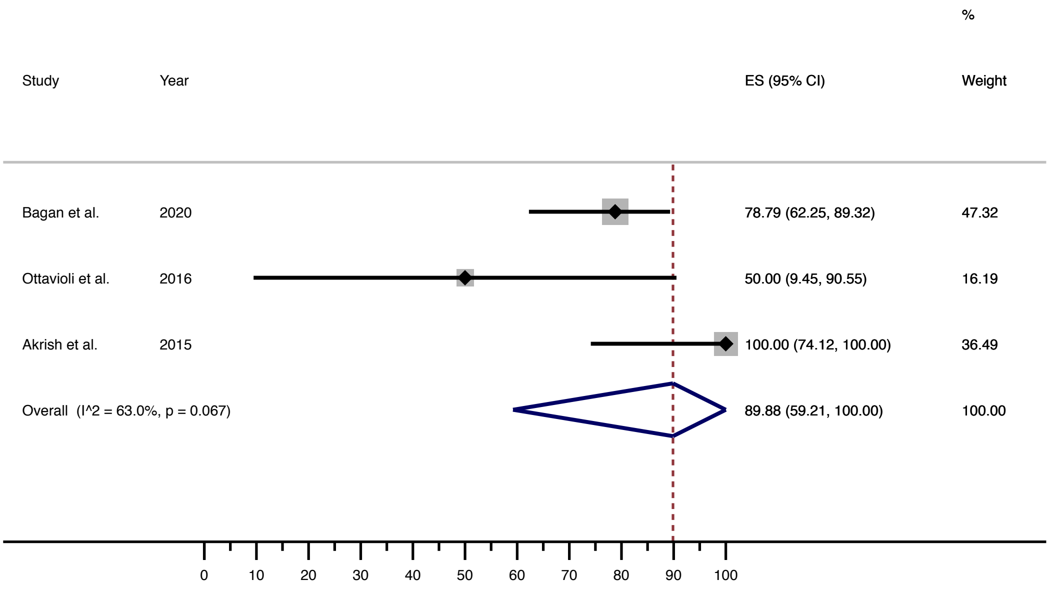

Abbreviations: ES, effect size (measured using pooled proportions); CI, confidence interval; PVL-OC, oral carcinomas developed in patients with proliferative verrucous leukoplakia.

6. Sensitivity analyses (leave-one-out method)

6.1. Table S2. Sensitivity analysis of studies included in the meta-analysis on mortality rate among patients with PVL-OC.

Table. Sensitivity analysis (leave-one-out method).				
**Study omitted**		**Effect size (%)**	**95% confidence intervals**	
1	Li et al. 2021	19.68	7.46	34.69
2	Favia et al. 2021	23.25	9.63	39.46
3	Bagan et al. 2020	16.92	6.57	29.68
4	Borgna et al. 2017	20.75	6.97	37.86
5	Flores et al. 2016	23.09	9.91	38.74
6	Ottavioli et al. 2016	20.77	8.28	35.94
7	Garcia-Pola et al. 2016	19.90	7.37	35.32
8	Owosho et al. 2015	22.58	9.67	37.98
9	Akrish et al. 2015	24.98	11.96	40.04
10	Ghazali et al. 2003	21.84	8.47	38.03
11	Fettig et al. 2000	21.59	8.32	37.69
12	Zakrzewska et al. 1996	21.39	7.94	37.83
13	Kahn et al. 1994	22.79	9.71	38.38
14	Hansen et al. 1985	18.65	5.99	34.63
**Combined**		PP=21.29	8.77	36.36

"Leave-one-out" sensitivity analysis of the meta-analysis of proportions, sequentially omitting one study at a time. Abbreviations: PP, pooled proportions.

6.2. Table S3. Sensitivity analysis of studies included in the meta-analysis on proportion of verrucous carcinomas among patients with PVL-OC.

Table. Sensitivity analysis (leave-one-out method).				
**Study omitted**		**Effect size (%)**	**95% confidence intervals**	
1	Li et al. 2021	35.48	18.88	53.62
2	Favia et al. 2021	29.03	15.83	43.80
3	Bagan et al. 2020	37.78	23.19	53.31
4	Koh and Kurago 2019	32.47	16.13	50.73
5	Upadhyaya et al. 2018	31.76	15.51	50.01
6	Villa et al. 2018	35.25	17.79	54.46
7	Borgna et al. 2017	32.77	15.78	51.81
8	Flores et al. 2016	34.08	17.47	52.45
9	Ottavioli et al. 2016	33.35	17.16	51.31
10	Garcia-Pola et al. 2016	34.08	17.47	52.45
11	Owosho et al. 2015	33.35	17.16	51.31
12	Akrish et al. 2015	33.00	16.23	51.71
13	Garcia-Chias et al. 2014	33.21	16.55	51.75
14	Morton et al. 2007	33.73	17.29	51.95
15	Klanrit et al. 2007	33.72	17.01	52.25
16	Campisi et al. 2005	35.64	18.40	54.52
17	Ghazali et al. 2003	32.44	16.00	50.83
18	Fettig et al. 2000	33.72	17.01	52.25
19	Kahn et al. 1994	32.55	16.38	50.60
20	Hansen et al. 1985	35.39	18.03	54.46
**Combined**		PP=33.66	17.58	51.43

"Leave-one-out" sensitivity analysis of the meta-analysis of proportions, sequentially omitting one study at a time. Abbreviations: PP, pooled proportions.

6.3. Table S4. Sensitivity analysis of studies included in the meta-analysis on proportion of oral squamous cell carcinomas among patients with PVL-OC.

**Table. Sensitivity analysis (leave-one-out method).**				
**Study omitted**		**Effect size (%)**	**95% confidence intervals**	
1	Li et al. 2021	70.60	50.84	87.63
2	Favia et al. 2021	74.92	55.50	90.99
3	Bagan et al. 2020	68.29	50.53	84.15
4	Koh and Kurago 2019	72.80	52.85	89.60
5	Upadhyaya et al. 2018	75.02	55.54	91.10
6	Villa et al. 2018	71.29	50.39	89.05
7	Borgna et al. 2017	74.39	54.24	91.03
8	Flores et al. 2016	70.37	50.52	87.51
9	Ottavioli et al. 2016	72.71	53.24	89.16
10	Garcia-Pola et al. 2016	72.09	52.18	88.99
11	Owosho et al. 2015	72.71	53.24	89.16
12	Akrish et al. 2015	69.44	49.65	86.70
13	Garcia-Chias et al. 2014	73.02	52.94	89.88
14	Morton et al. 2007	72.39	52.64	89.11
15	Klanrit et al. 2007	71.60	51.49	88.73
16	Campisi et al. 2005	70.86	50.27	88.47
17	Ghazali et al. 2003	71.41	51.24	88.61
18	Fettig et al. 2000	73.32	53.38	90.03
19	Kahn et al. 1994	70.60	50.84	87.63
20	Hansen et al. 1985	75.93	57.67	91.05
**Combined**		PP=72.21	52.95	88.64

"Leave-one-out" sensitivity analysis of the meta-analysis of proportions, sequentially omitting one study at a time. Abbreviations: PP, pooled proportions.

6.4. Table S5. Sensitivity analysis of studies included in the meta-analysis on proportion of well-differentiated oral squamous cell carcinomas among patients with PVL-OC.

**Table. Sensitivity analysis (leave-one-out method).**				
**Study omitted**		**Effect size (%)**	**95% confidence intervals**	
1	Favia et al. 2021	70.03	26.09	99.87
2	Borgna et al. 2017	71.36	24.39	100.00
3	Ottavioli et al. 2016	75.12	33.39	100.00
4	Garcia-Pola et al. 2016	73.98	28.26	100.00
5	Morton et al. 2007	79.83	34.19	100.00
6	Klanrit et al. 2007	81.07	34.08	100.00
7	Fettig et al. 2000	83.49	39.79	100.00
8	Zakrzewska et al. 1996	92.27	66.17	100.00
**Combined**		PP=78.41	37.24	100.00

"Leave-one-out" sensitivity analysis of the meta-analysis of proportions, sequentially omitting one study at a time. Abbreviations: PP, pooled proportions.

6.5. Table S6. Sensitivity analysis of studies included in the meta-analysis on proportion of T1/2 oral carcinomas among patients with PVL-OC.

**Table. Sensitivity analysis (leave-one-out method).**				
**Study omitted**		**Effect size (%)**	**95% confidence intervals**	
1	Ottavioli et al. 2016	100.00	74.12	100.00
2	Akrish et al. 2015	50.00	9.45	90.55
**Combined**		PP=99.93	81.37	100.00

"Leave-one-out" sensitivity analysis of the meta-analysis of proportions, sequentially omitting one study at a time. Abbreviations: PP, pooled proportions.

6.6. Table S7. Sensitivity analysis of studies included in the meta-analysis on proportion of N+ status among patients with PVL-OC.

**Table. Sensitivity analysis (leave-one-out method).**				
**Study omitted**		**Effect size (%)**	**95% confidence intervals**	
1	Ottavioli et al. 2016	2.58	0.00	19.45
2	Akrish et al. 2015	8.86	0.00	46.18
3	Fettig et al. 2000	0.00	0.00	10.32
**Combined**		PP=0.60	0.00	15.86

"Leave-one-out" sensitivity analysis of the meta-analysis of proportions, sequentially omitting one study at a time. Abbreviations: PP, pooled proportions.

6.7. Table S8. Sensitivity analysis of studies included in the meta-analysis on proportion of M+ status among patients with PVL-OC.

**Table. Sensitivity analysis (leave-one-out method).**				
**Study omitted**		**Effect size (%)**	**95% confidence intervals**	
1	Ottavioli et al. 2016	0.00	0.00	25.88
2	Akrish et al. 2015	50.00	9.45	90.55
**Combined**		PP=0.07	0.00	18.63

“Leave-one-out” sensitivity analysis of the meta-analysis of proportions, sequentially omitting one study at a time. Abbreviations: PP, pooled proportions.

6.8. Table S9. Sensitivity analysis of studies included in the meta-analysis on proportion of N+ status among patients with PVL-OC.

**Table. Sensitivity analysis (leave-one-out method).**				
**Study omitted**		**Effect size (%)**	**95% confidence intervals**	
1	Bagan et al. 2020	99.93	81.37	100.00
2	Ottavioli et al. 2016	86.74	74.26	95.96
3	Akrish et al. 2015	82.16	63.81	96.01
**Combined**		PP=89.88	59.21	100.00

“Leave-one-out” sensitivity analysis of the meta-analysis of proportions, sequentially omitting one study at a time. Abbreviations: PP, pooled proportions.

12. Analysis of small-study effects: Funnel plots

12.1 Funnel plot of mortality rate in patients with PVL-OC

Figure S13. A funnel plot of estimated transformed proportions against their standard errors, graphically representing the analysis of small-study effects on the mortality rate parameter in patients with PVL-OC.

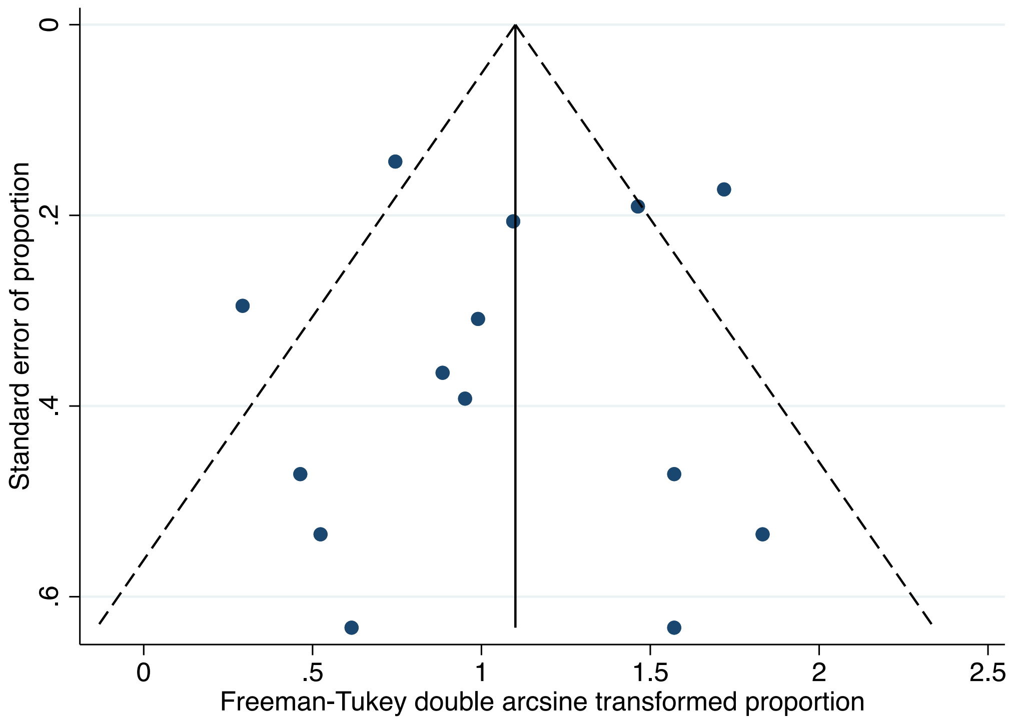

The black vertical line corresponds to the pooled estimated transformed prevalence. The two diagonal intermittent lines represent the pseudo-95% confidence interval. The blue circles represent the published studies.

12.2 Funnel plot of verrucous carcinomas in patients with PVL-OC

Figure S14. A funnel plot of estimated transformed proportions against their standard errors, graphically representing the analysis of small-study effects on the proportion of verrucous carcinomas in patients with PVL-OC.

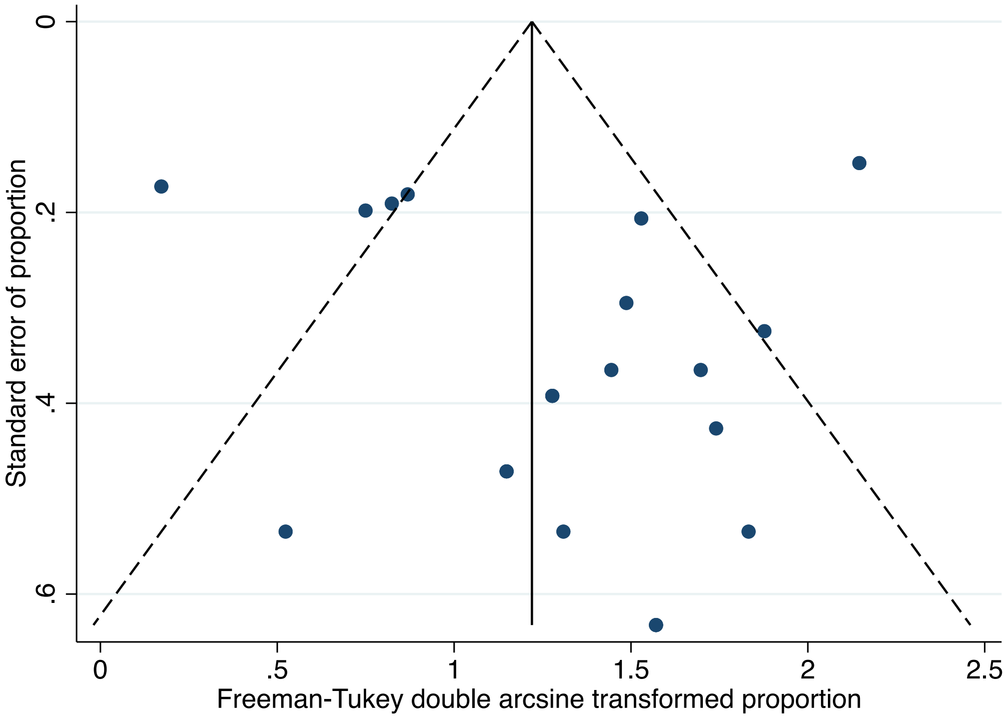

The black vertical line corresponds to the pooled estimated transformed prevalence. The two diagonal intermittent lines represent the pseudo-95% confidence interval. The blue circles represent the published studies.

12.3 Funnel plot of oral squamous cell carcinomas in patients with PVL-OC

Figure S15. A funnel plot of estimated transformed proportions against their standard errors, graphically representing the analysis of small-study effects on the proportion of oral squamous cell carcinomas in patients with PVL-OC.

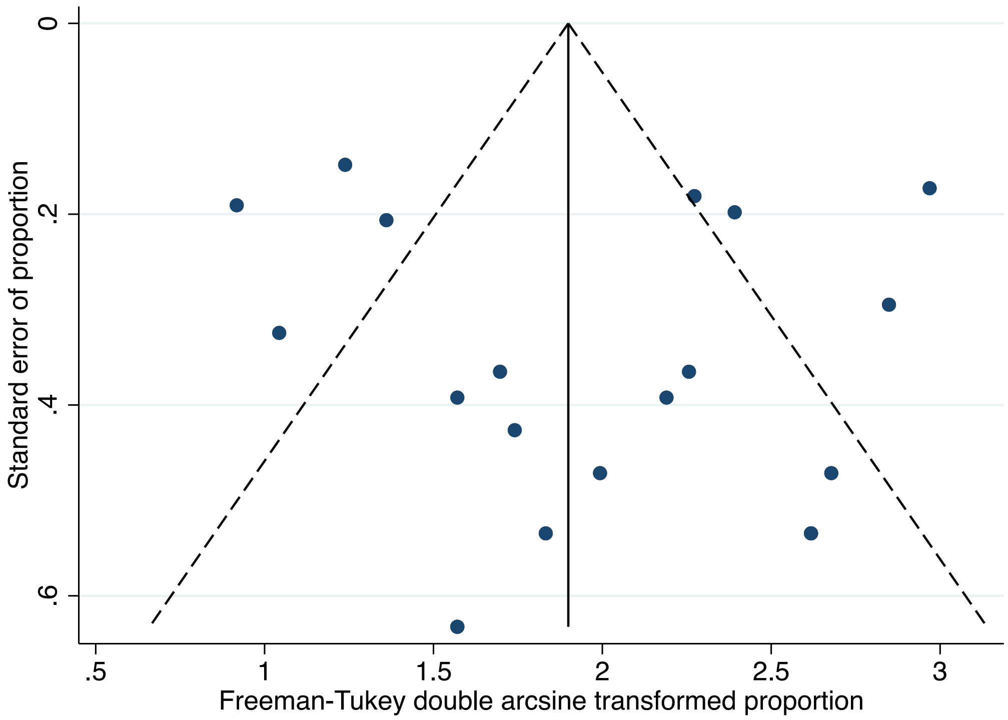

The black vertical line corresponds to the pooled estimated transformed prevalence. The two diagonal intermittent lines represent the pseudo-95% confidence interval. The blue circles represent the published studies.

12.4 Funnel plot of well differentiated oral squamous cell carcinomas in patients with PVL-OC

Figure S16. A funnel plot of estimated transformed proportions against their standard errors, graphically representing the analysis of small-study effects on the proportion of well-differentiated oral squamous cell carcinomas in patients with PVL-OC.

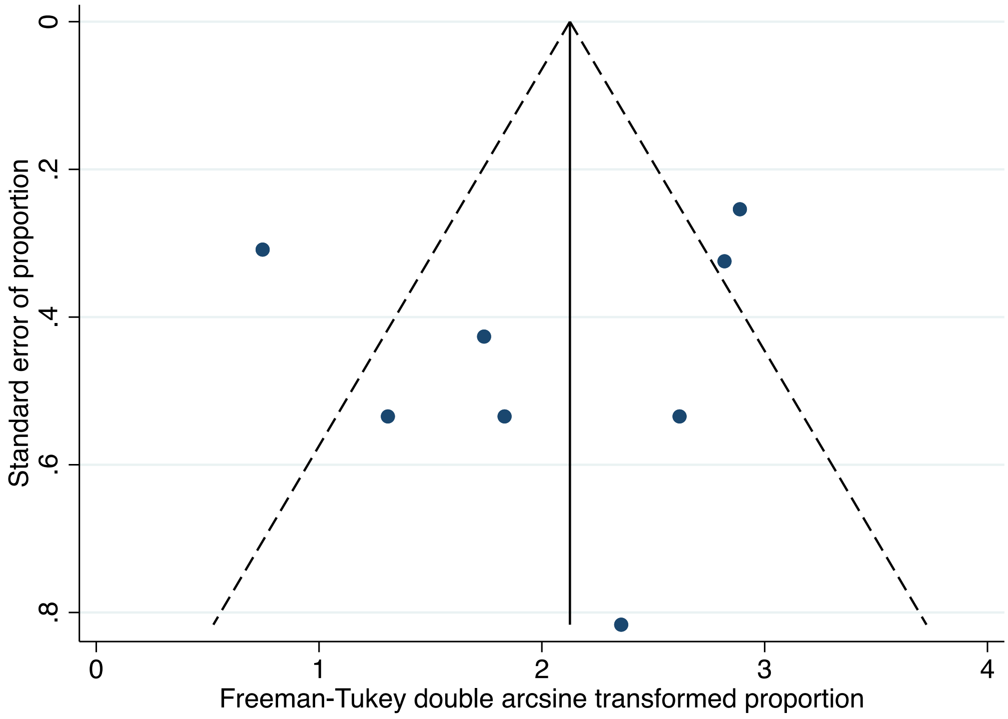

The black vertical line corresponds to the pooled estimated transformed prevalence. The two diagonal intermittent lines represent the pseudo-95% confidence interval. The blue circles represent the published studies.

12.5 Funnel plot of N status in patients with PVL-OC

Figure S17. A funnel plot of estimated transformed proportions against their standard errors, graphically representing the analysis of small-study effects on the proportion of N+ status cases among patients with PVL-OC.

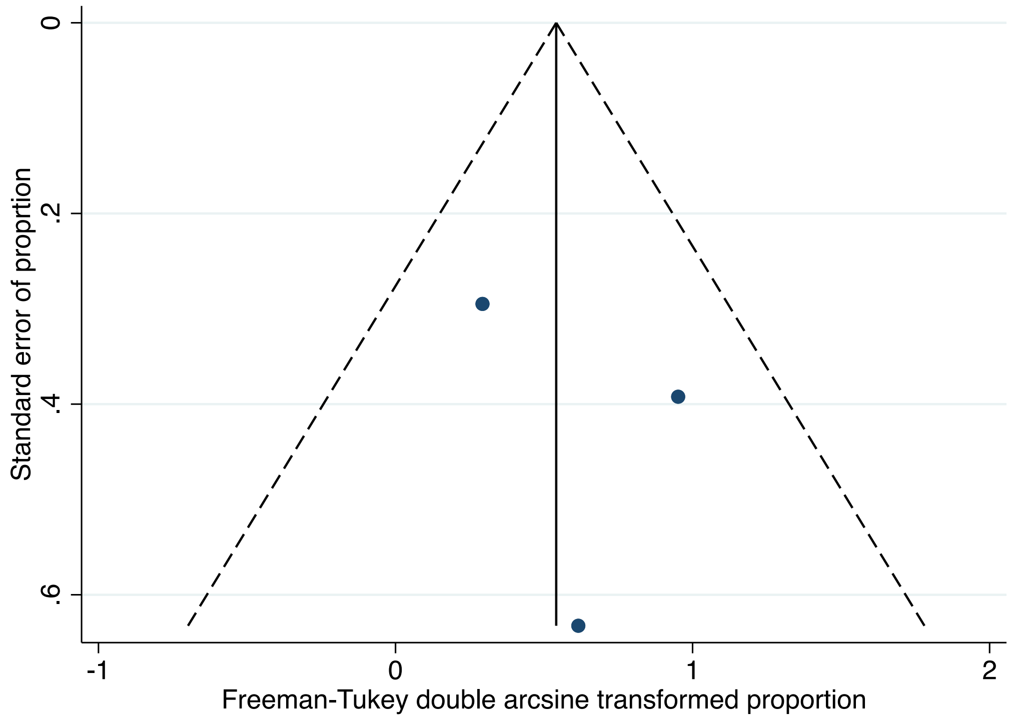

The black vertical line corresponds to the pooled estimated transformed prevalence. The two diagonal intermittent lines represent the pseudo-95% confidence interval. The blue circles represent the published studies.

12.6 Funnel plot of clinical stage in patients with PVL-OC

Figure S18. A funnel plot of estimated transformed proportions against their standard errors, graphically representing the analysis of small-study effects on the proportion of I/II-stage cases among patients with PVL-OC.

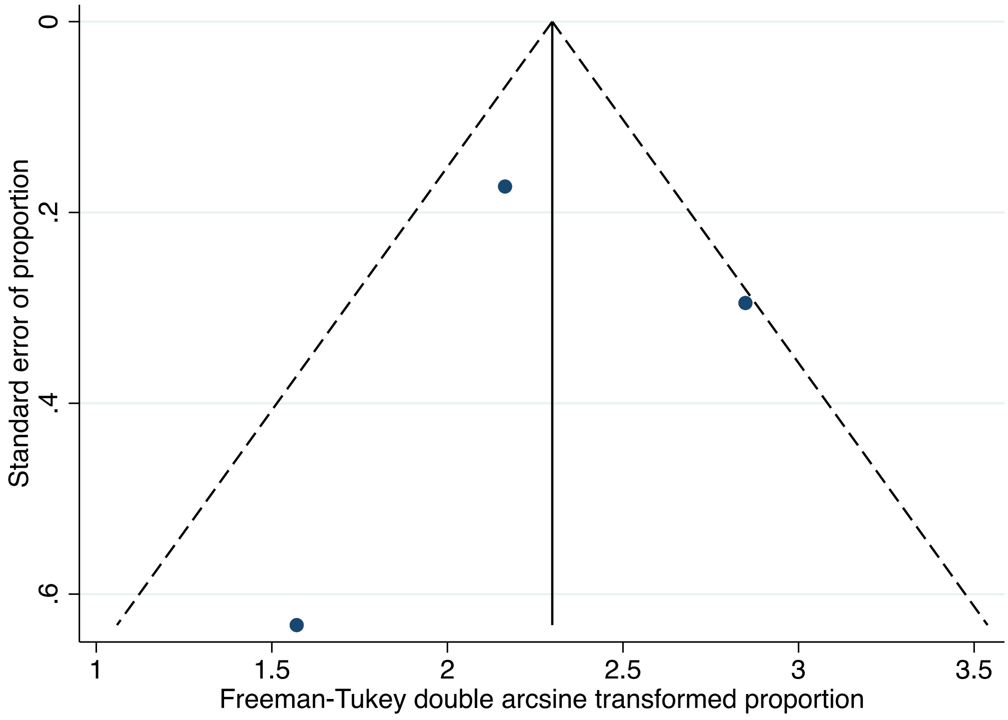

The black vertical line corresponds to the pooled estimated transformed prevalence. The two diagonal intermittent lines represent the pseudo-95% confidence interval. The blue circles represent the published studies.

13. List of excluded studies with reasons (n=22)

Overlapping population (n=7)

[1] Bagan J, Murillo-Cortes J, Leopoldo-Rodado M, Sanchis-Bielsa JM, Bagan L. Oral cancer on the gingiva in patients with proliferative leukoplakia: A study of 30 cases. J Periodontol 2019;90:1142–8. https://doi.org/10.1002/JPER.18-0620.

[2] Herreros-Pomares A, Llorens C, Soriano B, Zhang F, Gallach S, Bagan L, et al. Oral microbiome in Proliferative Verrucous Leukoplakia exhibits loss of diversity and enrichment of pathogens. Oral Oncol 2021;120:105404. https://doi.org/10.1016/j.oraloncology.2021.105404.

[3] Bagan L, Sáez GT, Tormos MC, Labaig-Rueda C, Murillo-Cortes J, Bagan J V. Salivary and serum interleukin-6 levels in proliferative verrucous leukoplakia. Clin Oral Investig 2016;20:737–43. https://doi.org/10.1007/s00784-015-1551-z.

[4] Bagán J V, Murillo J, Poveda R, Gavaldá C, Jiménez Y, Scully C. Proliferative verrucous leukoplakia: unusual locations of oral squamous cell carcinomas, and field cancerization as shown by the appearance of multiple OSCCs. Oral Oncol 2004;40:440–3. https://doi.org/10.1016/j.oraloncology.2003.10.008.

[5] Poveda-Roda R, Bagan J V, Jiménez-Soriano Y, Díaz-Fernández J-M, Gavaldá-Esteve C. Retinoids and proliferative verrucous leukoplakia (PVL). A preliminary study. Med Oral Patol Oral Cir Bucal 2010;15:e3-9. https://doi.org/10.4317/medoral.15.e3.

[6] Thomson PJ, Goodson ML, Smith DR. Treatment resistance in potentially malignant disorders-'Nature' or 'Nurture'…? J Oral Pathol Med 2017;46:902–10. https://doi.org/10.1111/jop.12641.

[7] Thomson PJ, Goodson ML, Cocks K, Turner JE. Interventional laser surgery for oral potentially malignant disorders: a longitudinal patient cohort study. Int J Oral Maxillofac Surg 2017;46:337–42. https://doi.org/10.1016/j.ijom.2016.11.001.

Lack of essential data (n=7)

[1] Qaisi M, Vorrasi J, Lubek J, Ord R. Multiple primary squamous cell carcinomas of the oral cavity. J Oral Maxillofac Surg 2014;72:1511–6. https://doi.org/10.1016/j.joms.2014.03.012.

[2] Upadhyaya JD, Fitzpatrick SG, Cohen DM, Bilodeau EA, Bhattacharyya I, Lewis JS, et al. Inter-observer Variability in the Diagnosis of Proliferative Verrucous Leukoplakia: Clinical Implications for Oral and Maxillofacial Surgeon Understanding: A

Collaborative Pilot Study. Head Neck Pathol 2020;14:156–65. https://doi.org/10.1007/s12105-019-01035-z.

[3] Haley JC, Hood AF, Mirowski GW. Proliferative verrucous leukoplakia with cutaneous involvement. J Am Acad Dermatol 1999;41:481–3. https://doi.org/10.1016/s0190-9622(99)70127-1.

[4] Kannan R, Bijur GN, Mallery SR, Beck FM, Sabourin CL, Jewell SD, et al. Transforming growth factor-alpha overexpression in proliferative verrucous leukoplakia and oral squamous cell carcinoma: an immunohistochemical study. Oral Surg Oral Med Oral Pathol Oral Radiol Endod 1996;82:69–74. https://doi.org/10.1016/s1079-2104(96)80379-9.

[5] García-López R, Moya A, Bagan J V, Pérez-Brocal V. Retrospective case-control study of viral pathogen screening in proliferative verrucous leukoplakia lesions. Clin Otolaryngol 2014;39:272–80. https://doi.org/10.1111/coa.12291.

[6] Femiano F, Gombos F, Scully C. Oral proliferative verrucous leukoplakia (PVL); open trial of surgery compared with combined therapy using surgery and methisoprinol in papillomavirus-related PVL. Int J Oral Maxillofac Surg 2001;30:318–22. https://doi.org/10.1054/ijom.2001.0066.

[7] Migliorati CA, Ranken R, Kaplan MJ, Silverman S. Reactivity of monoclonal antibodies 17.13 and 63.12 with 141 oral mucosal lesions. J Oral Pathol Med 1992;21:412–7. https://doi.org/10.1111/j.1600-0714.1992.tb01030.x.

No clinico-pathological or survival outcomes (n=2)

[1] Thomson PJ, Goodson ML, Smith DR. Potentially malignant disorders revisited-The lichenoid lesion/proliferative verrucous leukoplakia conundrum. J Oral Pathol Med 2018;47:557–65. https://doi.org/10.1111/jop.12716.

[2] McParland H, Warnakulasuriya S. Lichenoid morphology could be an early feature of oral proliferative verrucous leukoplakia. J Oral Pathol Med 2021;50:229–35. https://doi.org/10.1111/jop.13129.

Review (n=2)

[1] Beck-Mannagetta J, Hutarew G. [Squamous cell carcinoma and potentially malignant disorders of the oral mucosa]. Hautarzt 2009;60:859–65. https://doi.org/10.1007/s00105-009-1801-6.

[2] Grajewski S, Groneberg D. [Leukoplakia and erythroplakia--two orale precursor lesions]. Laryngorhinootologie 2009;88:666–72; quiz 673–5. https://doi.org/10.1055/s-0029-1241184.

Malignant transformation rate of 0% (n=1)

[1] Mehrotra D, Goel M, Kumar S, Pandey R, Ram H. Oral verrucous lesions: Controversies in diagnosis and management. J Oral Biol Craniofacial Res 2012;2:163–9. https://doi.org/10.1016/j.jobcr.2012.10.006.

Commentary (n=1)

[1] Brignardello-Petersen R. Proliferative verrucous leukoplakia and erythroplakia are probably the disorders with the highest rate of malignant transformation. J Am Dent Assoc 2020;151:e62. https://doi.org/10.1016/j.adaj.2020.01.035.

Letter (n=1)

[1] Olofsson J. Comment on “DNA ploidy in proliferative verrucous leukoplakia.” Oral Oncol 2007;43:621. https://doi.org/10.1016/j.oraloncology.2007.04.002.

Cross-sectional (n=1)

[1] Kresty LA, Mallery SR, Knobloch TJ, Li J, Lloyd M, Casto BC, et al. Frequent alterations of p16INK4a and p14ARF in oral proliferative verrucous leukoplakia. Cancer Epidemiol Biomarkers Prev 2008;17:3179–87. https://doi.org/10.1158/1055-9965.EPI-08-0574.
